# Transcriptome analysis reveals a molecular understanding of nicotinamide and butyrate sodium on meat quality of broilers under high stocking density

**DOI:** 10.1186/s12864-020-06827-0

**Published:** 2020-06-18

**Authors:** Yuqin Wu, Youli Wang, Dafei Yin, Tahir Mahmood, Jianmin Yuan

**Affiliations:** State Key Laboratory of Animal Nutrition, College of Animal Science and Technology, China Agricultural University, Beijing, 100193 China

**Keywords:** Stocking density, Broiler, Nicotinamide, Butyrate sodium, Transcriptome

## Abstract

**Background:**

In recent years, increased attention has been focused on breast muscle yield and meat quality in poultry production. Supplementation with nicotinamide and butyrate sodium can improve the meat quality of broilers. However, the potential molecular mechanism is not clear yet. This study was designed to investigate the effects of supplementation with a combination of nicotinamide and butyrate sodium on breast muscle transcriptome of broilers under high stocking density. A total of 300 21-d-old Cobb broilers were randomly allocated into 3 groups based on stocking density: low stocking density control group (L; 14 birds/m^2^), high stocking density control group (H; 18 birds/m^2^), and high stocking density group provided with a combination of 50 mg/kg nicotinamide and 500 mg/kg butyrate sodium (COMB; 18 birds/m^2^), raised to 42 days of age.

**Results:**

The H group significantly increased cooking losses, pH decline and activity of lactate dehydrogenase in breast muscle when compared with the L group. COMB showed a significant decrease in these indices by comparison with the H group (*P* < 0.05). The transcriptome results showed that key genes involved in glycolysis, proteolysis and immune stress were up-regulated whereas those relating to muscle development, cell adhesion, cell matrix and collagen were down-regulated in the H group as compared to the L group. In contrast, genes related to muscle development, hyaluronic acid, mitochondrial function, and redox pathways were up-regulated while those associated with inflammatory response, acid metabolism, lipid metabolism, and glycolysis pathway were down-regulated in the COMB group when compared with the H group.

**Conclusions:**

The combination of nicotinamide and butyrate sodium may improve muscle quality by enhancing mitochondrial function and antioxidant capacity, inhibiting inflammatory response and glycolysis, and promoting muscle development and hyaluronic acid synthesis.

## Background

Intensive stocking in the rapidly developing poultry industry worldwide has become a norm. However, high stocking density causes oxidative stress in broilers [1] and reduces the tenderness and increases the drip loss of breast muscle [2, 3]. Oxidation is one of the leading reasons for the deterioration of meat quality [4], and oxidative stress causes protein and lipid peroxidation as well as cellular damage [5, 6] which ultimately affects meat quality [7]. Nicotinamide (NAM) reduces oxidative stress and inhibits reactive oxygen species (ROS) production [8, 9]. Dietary supplementation with NAM has been observed to minimize the formation of carbonylated proteins in the liver of high-fat fed mice [10]. Butyrate sodium (BA) could also improve antioxidant capacity in a human study [11]. Further, the addition of BA can enhance the activities of superoxide dismutase and catalase and reduce the level of malondialdehyde in serum [12]. Butyrate treatment has been reported to decrease the levels of markers of oxidative stress and apoptosis in mice [13]. As treatment with NAM and BA both can elevate antioxidant capacity and muscle function, it may improve the muscle quality of broilers under high stocking density. Dietary supplementation with 60 mg/kg niacin (NAM precursor) reduces the drip loss of breast muscles in broilers [14]. Dietary supplementation with BA can increase broiler weight, decrease abdominal fat percentage [15], and reduce intramuscular fat content [16].

Mitochondrial biogenesis has previously been associated with preservation of muscle mass and beneficial effects on metabolism [17]. Peroxisome proliferator-activated receptor-γ coactivator 1α (PGC1α) is a crucial regulator of mitochondrial biogenesis. Replenishment with nicotinamide adenine dinucleotide (NAD) induces mitochondrial biogenesis by increasing PGC1α expression [18, 19]. NAM is the primary source of NAD which is obtained through the salvage pathway. As a precursor of NAD, treatment with NAM also enhances PGC-1α expression [20]. Impaired intramuscular NAD synthesis compromises skeletal muscle mass and strength over time, which can be quickly restored with an oral NAD precursor [21]. Besides, NAD biosynthesis alleviates muscular dystrophy in a zebrafish model [22] and promotes muscle function in *Caenorhabditis elegans* [23]. Addition of niacin (precursor of NAM) has been reported to increase the number of oxidative type I fibres in skeletal muscles of growing pigs [24] and induce type II to type I muscle fibre transition in sheep [25]. Further, supplementation with butyrate increases mitochondrial function and biogenesis of skeletal muscle in mice and rats [26, 27]. Further, the intake of BA increases the percentage of type 1 fibres [26, 28] and muscle fibre cross-sectional area in skeletal muscle [13].

Although supplementation with NAM or BA alone can elevate antioxidant capacity and improve the meat quality of broilers, the effect of combined supplementation with NAM and BA on the meat quality of broilers is not clear yet. Therefore, we performed transcriptome sequencing of broiler breast muscles to elucidate the molecular mechanism of the effect of feeding density and nutrient regulation on meat quality.

## Results

### Production performance and meat quality

**Fig. 1:**
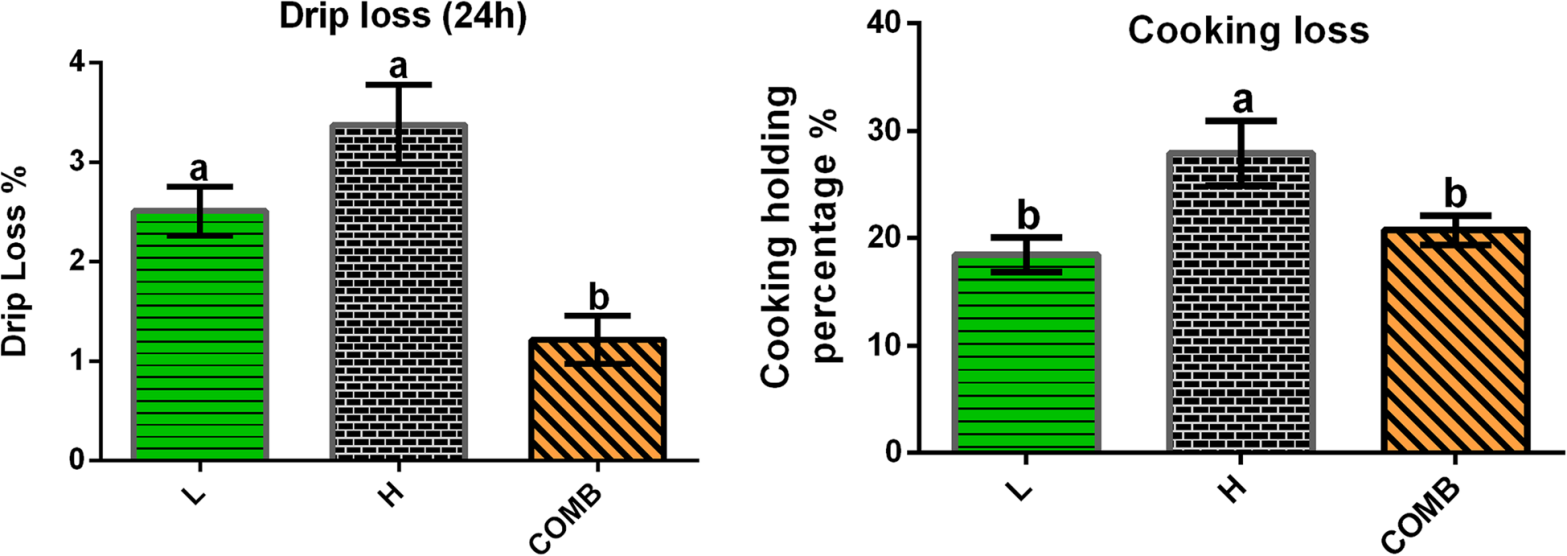
Water holding capacity of breast muscle. Data are shown as the means ± SEM. Different letters a, b indicate that there are significant differences (*P* < 0.05) among these groups. L, low stocking density (14 birds/m^2^); H, high stocking density (18 birds/m^2^); COMB, combination of NAM and BA (18 birds/m^2^)

There is no significant difference among the H, L and COMB group in corresponding to FI, BW, BWG and FCR (*P* > 0.05) (Table 1). Compared with the L group, the H group showed significantly increased cooking loss of breast muscle (*P* < 0.05). The COMB group showed decreased cooking loss compared with the H group (*P* < 0.05). Besides, the drip loss in the COMB group was lower than that in the L group, as well (*P* < 0.05) (Fig. 1).

**Table 1:** Production performance of broilers

	L	H	COMB	SEM	*P*-value
FI /g	2843	2844	2844	27.8	1.000
BW /g	2788	2745	2773	25.6	0.802
BWG /g	1610	1533	1567	23.6	0.439
FCR	1.77	1.86	1.82	0.02	0.188

Production performance included FI (feed intake), BW (body weight), BWG (body weight gain) and FCR (feed conversion ratio)

**Fig. 2:**
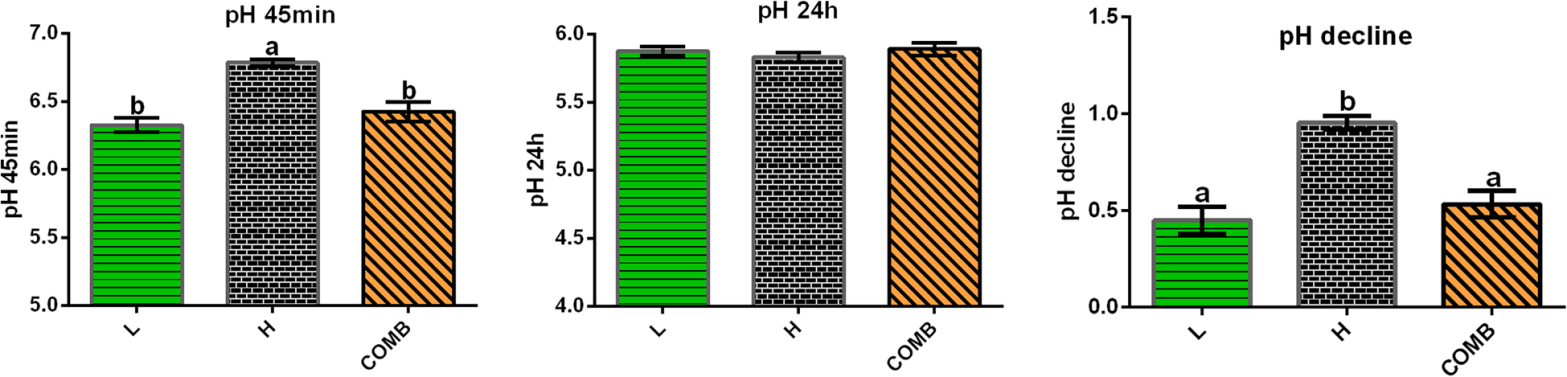
The pH values of breast muscle. Data are shown as the means ± SEM. Different letters a, b indicate that there are significant differences (*P* < 0.05) among these groups. L, low stocking density (14 birds/m^2^); H, high stocking density (18 birds/m^2^); COMB, combination of NAM and BA (18 birds/m^2^)

The 45-min pH value in the H group was higher than that in the other 2 groups (*P* < 0.05) while there was no significant difference in 24-h pH values among the groups. Thus, the pH decline during 45 min to 24 h in the H group was significantly higher than that in the other 2 groups, indicating that the H group had rapid pH drop rate, which was attenuated in the COMB group under high stocking density (Fig. 2).

### Anti-oxidant capacity

The stocking density significantly altered the activity of LDH (*P* = 0.022). The activity of LDH in the H group was higher (*P* < 0.05) than that in the L group. The COMB group had significantly decreased (*P* < 0.05) activity of LDH when compared with the H group. However, stocking density had no significant effect on the activities of CK, T-AOC, MDH, anti-superoxide anion and the content of hydroxyproline (Table 2).

**Table 2:** Enzyme activities of the breast muscle

	L	H	COMB	SEM	*P*-value
CK /U/mgprot	2.51	2.41	2.25	0.12	0.702
LDH /U/gprot	450.38^a^	724.10^b^	383.22^a^	56.74	0.022
T-AOC /U/mgprot	100.81	82.17	86.01	8.25	0.650
MDH /U/mgprot	1.37	1.21	1.53	0.08	0.252
Anti-superoxide anion /U/gprot	10.30	9.32	10.39	0.39	0.489
Hydroxyproline /μg/mg	155.56	164.22	172.01	8.51	0.755

### RNA sequencing data and differentially expressed genes (DEGs)

**Fig. 3:**
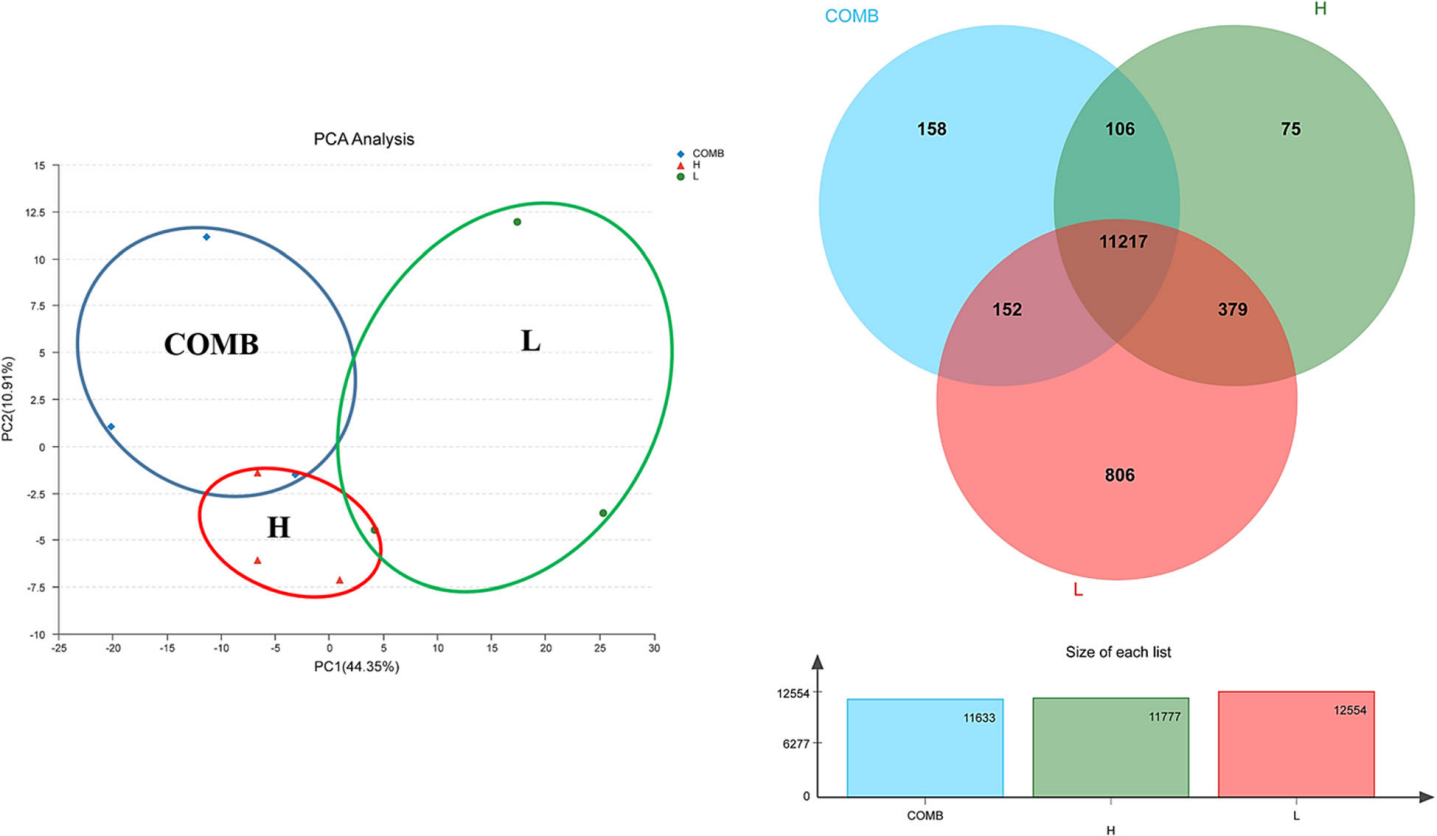
Principal Component Analysis (PCA) and Wayne (VEEN) analysis of gene sets. For the PCA graph, the distance between each sample point represents the distance of the sample. The closer the distance means higher the similarity between samples; for the VEEN graph, the numbers inside the circle represents the sum of the number of expressed genes in the group. The crossover region represents the number of consensus expressed genes for each group

**Fig. 4:**
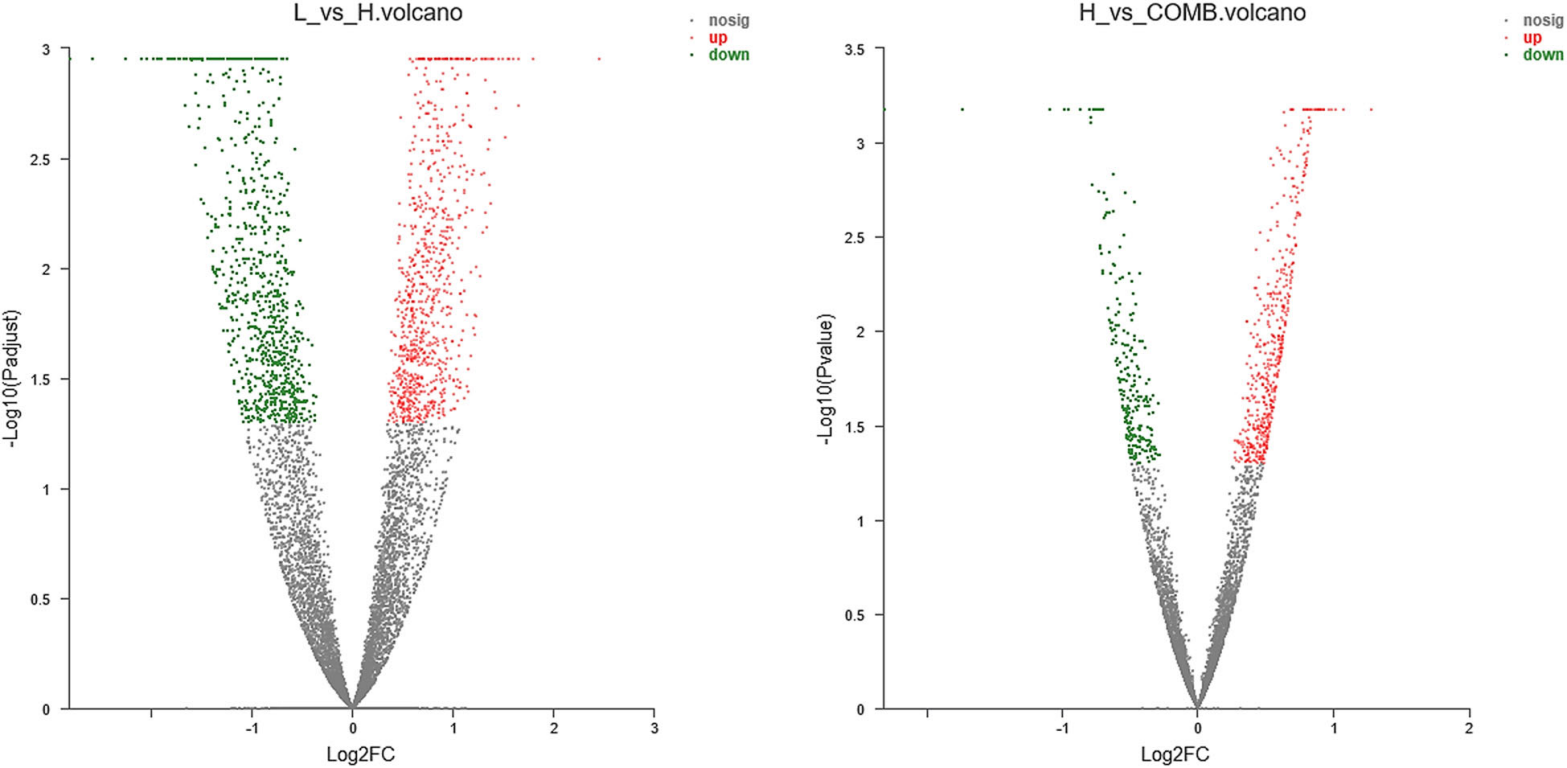
Volcanic map of differential expression genes. The abscissa is the fold change of the gene expression difference between the two samples and the ordinate is the statistical test value of the gene expression. Each dot in the figure represents a specific gene, the red dot indicates a significantly up-regulated gene, the green dot indicates a significantly down-regulated gene, and the grey dot is a non-significant differential gene

In the principal component analysis (PCA), there was a clear divergence among the H, L and COMB groups. In the Venn diagram, the number of identified genes in the H, L and COMB were 11,777, 12,554 and 11,633, respectively (Fig. 3). Compared with the H group, the number of DEGs in the L group and COMB group were 3752 and 773, respectively (Fig. 4).

**Fig. 5:**
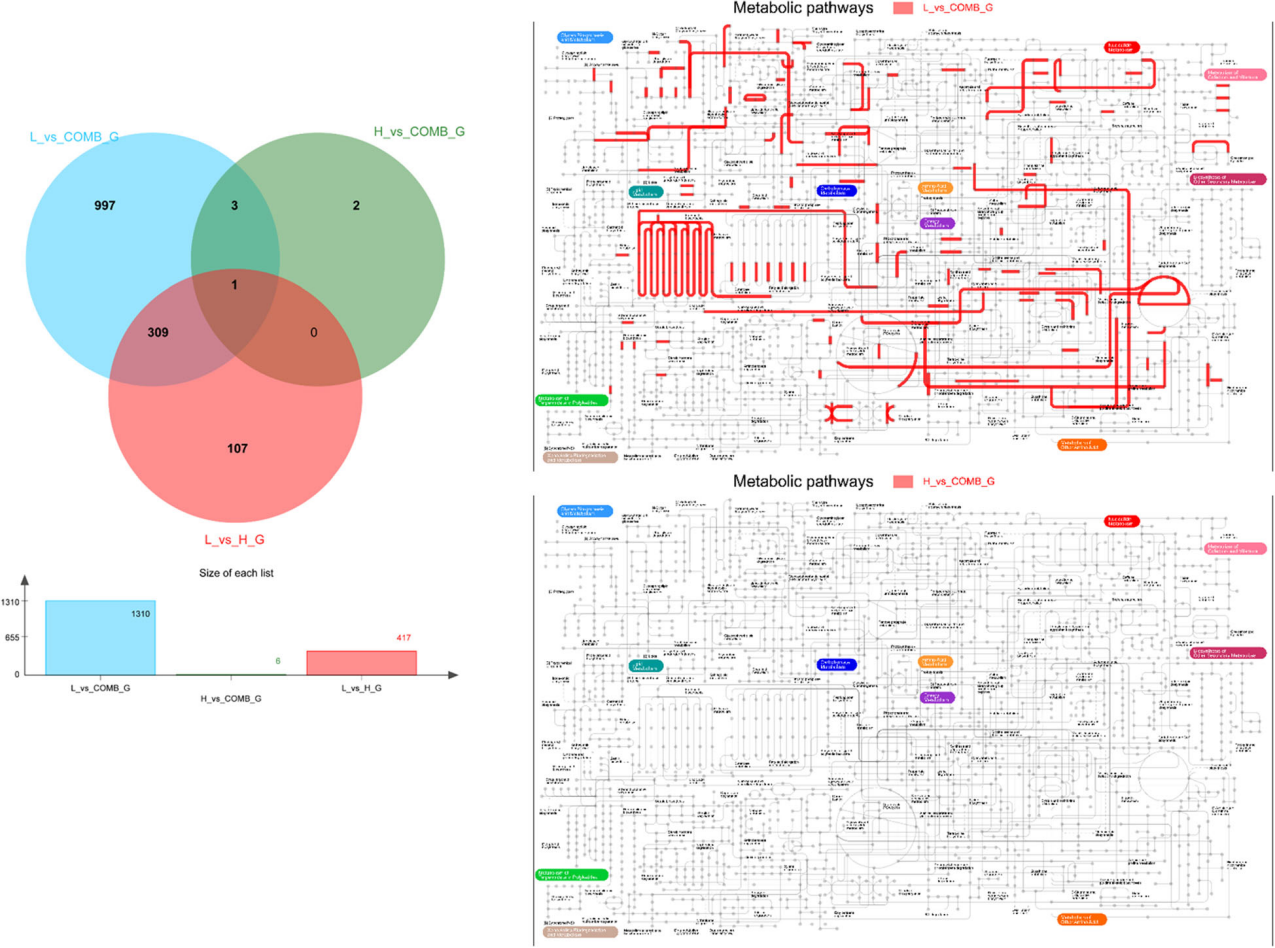
The Veen diagram and the map of Kyoto Encyclopedia of Genes and Genomes (KEGG) pathways analysis of gene sets. For VEEN diagram: the sum of all the numbers inside the circle represents the total gene of the set. The number, circle intersection area represents the number of shared genes among the gene sets. For the map of KEGG metabolic pathway, the red represents the pathway of the common annotation of the genes in the gene sets of two groups. We thank Kanehisa Laboratories for providing the copyright permission of KEGG pathway maps [29]

The gene sets were produced by DEGS. From Venn analysis of genes sets, we found that there were 1310 genes shared in common between the COMB group and the L group. Nevertheless, there were only 6 genes owed by both the COMB group and the H group. Similarly, from the iPath map of metabolic pathways, there were a total of 830 pathways annotated in common. In contrast, there was only 1 pathway owed by both the COMB group and the H group (Fig. 5).

### Up-regulated genes in the H group

**Fig. 6:**
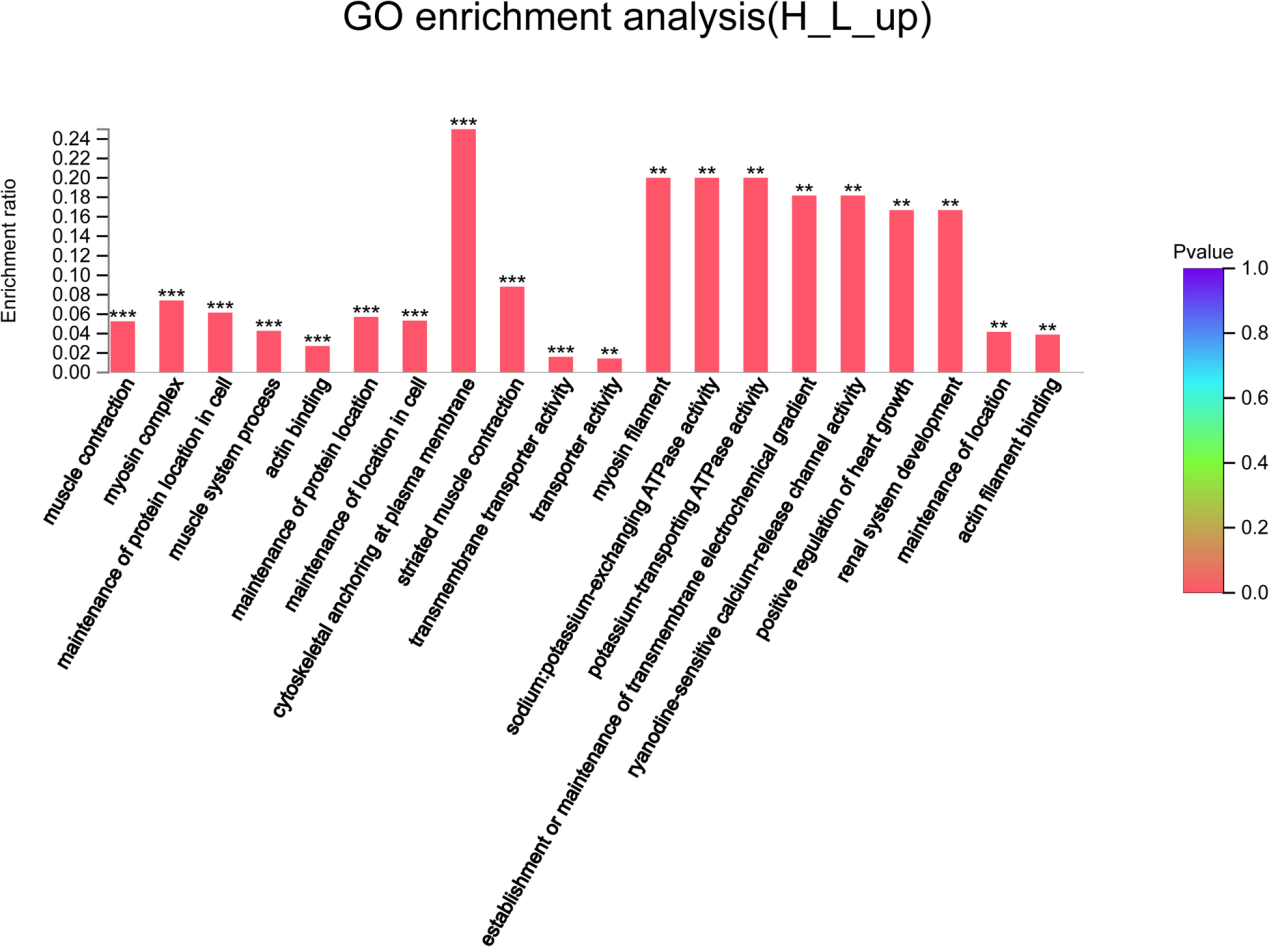
GO enrichment analysis of up-regulated genes in the H group. The abscissa indicates the GO term, and the ordinate indicates the enrichment ratio. “*“means *P* < 0.05, “**“means *P* < 0.01 and “***” means *P* < 0.001

Compared with those in the L group, a total of 1894 genes were up-regulated in the H group (Fig. 4), which were mainly involved in muscle contraction, cell localization, ion transport, lipid metabolism, glycolysis, proteolysis, and immune stress (Fig. 6).

Muscle contraction-related pathways were enriched in the H group. They involved vital genes including MYLK2, NOS1, TMOD4, and Six1 (Table 3). The H group was enriched for cell-localization-related genes such as KEAP1, CDKN1A, ERBB4, and TMOD4 (Table 3). Additionally, high-density up-regulated ion and amino acid transport-related genes included KCNJ12, KCNA7, SLC38A3 and SLC38A4, which are involved in ion transmembrane transport and transporter activity (Table 4). High-density enriched glycolysis-related pathways included fructose metabolism, fructose-2,6-diphosphate 2-phosphatase activity, and fructose 2,6-diphosphate metabolism (Table 5). The lipid metabolism-related genes such as MID1IP1, ACACB and Lpin1 were up-regulated in H group, which are involved in lipid synthesis and lipid oxidation (Table 5).

**Table 3:** Muscle contraction and cell location related pathways

GO ID	Term Type	Description	***P***-value	Genes
**Muscle contraction related pathways**				
GO:0044449	CC	contractile fiber part	0.026498	NOS1; TMOD4
GO:0006936	BP	muscle contraction	0.000194	MYLK2; NOS1
GO:0006941	BP	striated muscle contraction	0.000908	MYLK2; NOS1
GO:0003012	BP	muscle system process	0.00051	MYLK2; NOS1
GO:0051015	MF	actin filament binding	0.002704	TMOD4
GO:0003779	MF	actin binding	0.000614	TMOD4
GO:0008092	MF	cytoskeletal protein binding	0.033316	TMOD4
GO:0004687	MF	myosin light chain kinase activity	0.022364	MYLK2
**Cell location related pathways**				
GO:0051235	BP	maintenance of location	0.002093	KEAP1
GO:0051651	BP	maintenance of location in cell	0.000837	KEAP1
GO:0045185	BP	maintenance of protein location	0.000645	KEAP1
GO:0032507	BP	maintenance of protein location in cell	0.000486	KEAP1
GO:1900180	BP	regulation of protein localization to nucleus	0.032179	KEAP1; CDKN1A; ERBB4
GO:2000010	BP	positive regulation of protein localization to cell surface	0.044234	ERBB4
GO:0042306	BP	regulation of protein import into nucleus	0.018345	KEAP1; CDKN1A; ERBB4
GO:1904589	BP	regulation of protein import	0.018837	KEAP1; CDKN1A; ERBB4

**Table 4:** Ion transport related pathways

GO ID	Term Type	Description	***P-***value	Genes
**Ion transport related pathways**				
GO:0030001	BP	metal ion transport	0.015075	KCNJ12
GO:0002028	BP	regulation of sodium ion transport	0.017458	NOS1
GO:0051365	BP	cellular response to potassium ion starvation	0.011244	SLC38A3
GO:0006813	BP	potassium ion transport	0.030866	KCNJ12
GO:0034220	BP	ion transmembrane transport	0.015681	SLC38A4; SLC38A3; KCNJ12
GO:0010107	BP	potassium ion import	0.004526	KCNJ12
GO:0006813	BP	potassium ion transport	0.030866	KCNJ12
GO:0098655	BP	cation transmembrane transport	0.024337	SLC38A3; KCNJ12
GO:0006812	BP	cation transport	0.027707	SLC38A3; KCNJ12
GO:0098662	BP	inorganic cation transmembrane transport	0.046453	KCNJ12
GO:0015075	MF	ion transmembrane transporter activity	0.008902	KCNA7; SLC38A4; SLC38A3
GO:0046873	MF	metal ion transmembrane transporter activity	0.007993	KCNJ12
GO:0008324	MF	cation transmembrane transporter activity	0.01451	SLC38A3; KCNJ12
GO:0022890	MF	inorganic cation transmembrane transporter activity	0.022537	KCNJ12
GO:0005261	MF	cation channel activity	0.045897	KCNJ12
GO:0005216	MF	ion channel activity	0.03925	KCNA7; KCNJ12
GO:0015276	MF	ligand-gated ion channel activity	0.026498	KCNJ12
GO:0015079	MF	potassium ion transmembrane transporter activity	0.029581	KCNJ12

**Table 5:** Glycolysis and lipid metabolism related pathways

GO ID	Term Type	Description	***P-***value	Genes
**Glycolysis related pathways**				
GO:0006000	BP	fructose metabolic process	0.038812	PFKFB1
GO:0004331	MF	fructose-2,6-bisphosphate 2-phosphatase activity	0.01682	PFKFB1
GO:0003873	MF	6-phosphofructo-2-kinase activity	0.022364	PFKFB1
GO:0050308	MF	sugar-phosphatase activity	0.038812	PFKFB1
GO:0008443	MF	phosphofructokinase activity	0.038812	PFKFB1
GO:0006003	BP	fructose 2,6-bisphosphate metabolic process	0.022364	PFKFB1
**Lipid metabolism related pathways**				
GO:0003989	MF	acetyl-CoA carboxylase activity	0.044234	ACACB
GO:0019217	BP	regulation of fatty acid metabolic process	0.016548	MID1IP1; ACACB
GO:0046949	BP	fatty-acyl-CoA biosynthetic process	0.03336	ACACB
GO:0019432	BP	triglyceride biosynthetic process	0.03336	Lpin1
GO:0046463	BP	acylglycerol biosynthetic process	0.038812	Lpin1
GO:0046460	BP	neutral lipid biosynthetic process	0.038812	Lpin1
GO:0046322	BP	negative regulation of fatty acid oxidation	0.01682	ACACB
GO:0031998	BP	regulation of fatty acid beta-oxidation	0.044234	ACACB
GO:0031999	BP	negative regulation of fatty acid beta-oxidation	0.011244	ACACB
GO:0045723	BP	positive regulation of fatty acid biosynthetic process	0.027877	MID1IP1
GO:0010884	BP	positive regulation of lipid storage	0.044234	ACACB
GO:2001295	BP	malonyl-CoA biosynthetic process	0.011244	ACACB
GO:2001293	BP	malonyl-CoA metabolic process	0.01682	ACACB
GO:0010565	BP	regulation of cellular ketone metabolic process	0.047727	MID1IP1; ACACB

Stress response pathways including non-biologically stimulated cellular responses, extracellular stimuli response and nutritional level response were also enriched in the H group. Furthermore, high-density up-regulated proteolysis-related genes include TINAG, USP24, OTUD1, KEAP1, KLHL34, and SMCR8. Also, high-density enriched immune pathways include the regulation of host defence responses to viruses and prostaglandin receptor-like binding (Table 6).

**Table 6:** Proteolysis, immune and stress related pathways

GO ID	Term Type	Description	***P-***value	Genes
**Proteolysis related pathways**				
GO:0008234	MF	cysteine-type peptidase activity	0.032179	TINAG; USP24; OTUD1
GO:0031463	CC	Cul3-RING ubiquitin ligase complex	0.028791	KEAP1; KLHL34
GO:0010499	BP	proteasomal ubiquitin-independent protein catabolic process	0.03336	KEAP1
GO:0010508	BP	positive regulation of autophagy	0.034688	SMCR8
GO:1902902	BP	negative regulation of autophagosome assembly	0.03336	SMCR8
GO:1901096	BP	regulation of autophagosome maturation	0.011244	SMCR8
GO:1901098	BP	positive regulation of autophagosome maturation	0.011244	SMCR8
**Immune and stress related pathways**				
GO:0031867	MF	EP4 subtype prostaglandin E2 receptor binding	0.005638	FEM1A
GO:0031862	MF	prostanoid receptor binding	0.005638	FEM1A
GO:0050691	BP	regulation of defense response to virus by host	0.031097	ALKBH5; ALPK1
GO:0002230	BP	positive regulation of defense response to virus by host	0.026558	ALKBH5; ALPK1
GO:0071214	BP	cellular response to abiotic stimulus	0.042948	CDKN1A; SLC38A3
GO:0009991	BP	response to extracellular stimulus	0.022488	ACACB; CDKN1A; SLC38A3
GO:0031667	BP	response to nutrient levels	0.018345	ACACB; CDKN1A; SLC38A3

In Kyoto Encyclopedia of Genes and Genomes (KEGG) enrichment analysis, genes involved in calcium signalling pathway (RYR), inflammatory mediator regulation of RTP channels (PLA2) and chemokine signalling pathway (SOS) (Fig. S1, S2 and S3) were enriched in the H group.

### Down-regulated genes in the H group

**Fig. 7:**
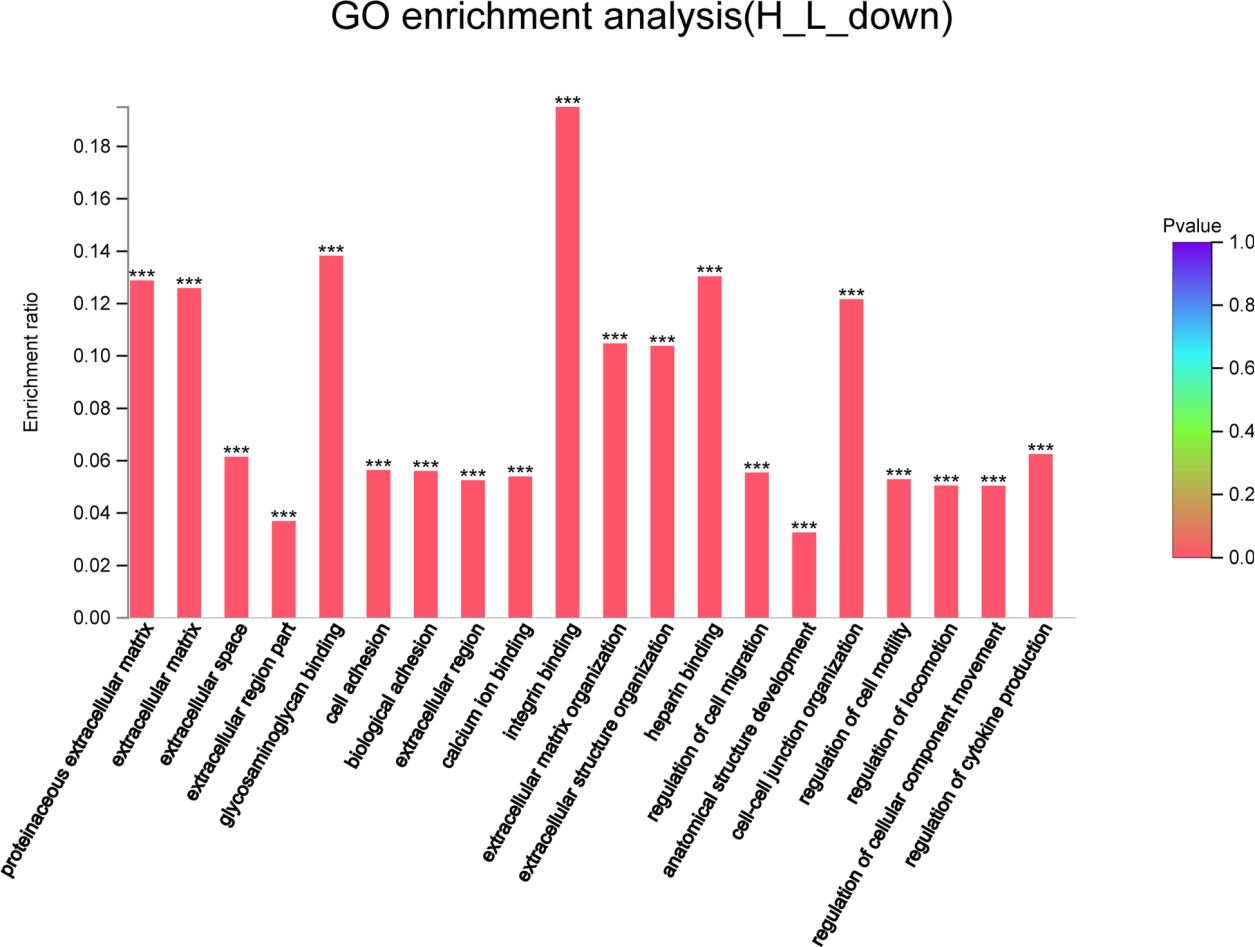
GO enrichment analysis of down-regulated genes in the H group. The abscissa indicates the GO term, and the ordinate indicates the enrichment ratio. “*“means *P* < 0.05, “**“means *P* < 0.01 and “***” means *P* < 0.001

Compared with those in the L group, a total of 1858 genes were down-regulated in the H group (Fig. 4), which were involved in cell adhesion, cell matrix, and cell migration, etc. (Fig. 7).

The genes involved in muscle development include muscle fibre assembly and binding (LMOD2, MYOZ2 and ACTN1, etc.) and muscle fibre development (DSG2, LMOD2 and FSCN1, etc.), which were down-regulated in H group (Table 7). High-density also down-regulated genes related to cell-matrix pathways such as MMP9, FBLN1, THBS4, and VCAN. High-density also down-regulated collagen synthesis and collagen binding related genes including ADAMTS3, ADAMTS14, COL1A2, and LUM (Table 8). Besides, the adhesion-associated genes including DSG2, CSTA, THY1, TGFBI, NOV, CDH11 and FN1 were diminished. Additionally, antioxidant genes including MGST2, PTGS2, NCF1, SOD3, and CYBB were also down-regulated (Table 9).

**Table 7:** Muscle development related pathway

GO ID	Term Type	Description	***P-***value	Genes
**Muscle development related pathways**				
GO:0030239	BP	myofibril assembly	0.021003	LMOD2; MYOZ2
GO:0043205	CC	fibril	0.008763	FN1; LTBP1
GO:0045214	BP	sarcomere organization	0.045011	LMOD2; ACTN1
GO:0051017	BP	actin filament bundle assembly	9.31E-05	LIMA1; ACTN1; DPYSL3; FSCN1
GO:0061572	BP	actin filament bundle organization	0.00013	LIMA1; ACTN1; DPYSL3; FSCN1
GO:0007015	BP	actin filament organization	0.001785	LIMA1; LMOD2; ACTN1; DPYSL3; FSCN1
GO:0030036	BP	actin cytoskeleton organization	0.002238	LMOD2; MYOZ2; Fgf7; ACTN1; MYL6; CNN2; DOCK2; FSCN1
GO:0031032	BP	actomyosin structure organization	0.001641	LMOD2; MYOZ2; ACTN1; MYL6; CNN2
GO:0003779	MF	actin binding	0.000306	MYH15; LIMA1; LMOD2; MYOZ2; ACTN1; MYL6; CNN2; MYL3; FSCN1
GO:0005523	MF	tropomyosin binding	0.006889	LMOD2; S100A6
GO:0070051	MF	fibrinogen binding	0.016237	FBLN1
GO:0050436	MF	microfibril binding	0.032211	LTBP1
GO:0060537	BP	muscle tissue development	0.029507	DSG2; EYA2; BMP5; ITGA8
GO:0032970	BP	regulation of actin filament-based process	0.033864	DSG2; LIMA1; LMOD2; WNT11; SERPINF2; FSCN1; F2RL1
GO:0030029	BP	actin filament-based process	0.003744	LMOD2; MYOZ2; Fgf7; ACTN1; MYL6; CNN2; DOCK2; FSCN1
GO:0014883	BP	transition between fast and slow fiber	0.047928	TNNI1
GO:1902724	BP	positive regulation of skeletal muscle satellite cell proliferation	0.047928	HGF

**Table 8:** Cellular matrix and collagen related pathway

GO ID	Term Type	Description	***P-***value	Genes
**Cellular matrix related pathways**				
GO:0030198	BP	extracellular matrix organization	1.05E-06	MMP9; TGFBI; ABI3BP; POSTN; FBLN1, etc
GO:0044420	CC	extracellular matrix component	2.83E-05	COL1A2; FN1; THBS2; THBS4; LTBP1, etc
GO:0005578	CC	proteinaceous extracellular matrix	1.71E-11	FN1; THBS2; ADAMTS3; ADAMTS14; COL12A1, etc
GO:0005614	CC	interstitial matrix	0.013106	FN1; ABI3BP
GO:0043062	BP	extracellular structure organization	1.15E-06	MMP9; TGFBI; ABI3BP; ADAMTS14; POSTN, etc
GO:0005201	MF	extracellular matrix structural constituent	0.017449	MGP; VCAN; FBLN1
GO:0031232	CC	extrinsic component of external side of plasma membrane	0.032211	SERPINE2
GO:0019897	CC	extrinsic component of plasma membrane	0.000874	SERPINE2; S100A6; RGS1; KCNAB1
GO:1990430	MF	extracellular matrix protein binding	0.047928	ITGB8
**Collagen related pathways**				
GO:0032964	BP	collagen biosynthetic process	0.047928	ADAMTS3
GO:0032963	BP	collagen metabolic process	0.027114	MMP9; ADAMTS3
GO:0010712	BP	regulation of collagen metabolic process	0.023978	SERPINF2; FAP
GO:0010710	BP	regulation of collagen catabolic process	0.032211	FAP
GO:0030199	BP	collagen fibril organization	0.000216	ADAMTS14; SFRP2; LUM; SERPINF2
GO:0005518	MF	collagen binding	0.00265	TGFBI; ABI3BP; COMP; LUM
GO:0005540	MF	hyaluronic acid binding	0.037432	TNFAIP6; VCAN
GO:0005581	CC	collagen trimer	0.000699	COL1A2; COL12A1; COLEC12; LUM; COL14A1
GO:0005583	CC	fibrillar collagen trimer	0.001541	COL1A2; LUM
GO:0005539	MF	glycosaminoglycan binding	3.59E-09	MDK; SLIT3; NOV; SERPINE2; JCHAIN, etc
GO:1901617	BP	organic hydroxy compound biosynthetic process	0.033592	NR4A2; PLTP; LCAT; AKR1D1

**Table 9:** Cell adhesion and antioxidant related pathway

GO ID	Term Type	Description	***P-***value	Genes
**Cell adhesion related pathways**				
GO:0007155	BP	cell adhesion	3.1E-08	DSG2; TGFBI; NOV; FN1; THBS2; COMP, etc
GO:0098609	BP	cell-cell adhesion	0.030671	DSG2; CSTA; NOV; CDH11; THBS4; BMP5, etc
GO:0007160	BP	cell-matrix adhesion	0.02187	FN1; ITGB8; ITGA8
GO:0050839	MF	cell adhesion molecule binding	0.000141	DSG2; THY1; TGFBI; NOV; FN1; THBS4, etc
GO:0005911	CC	cell-cell junction	0.001091	DSG2; CD3E; GJA1; NOV; ABCB11; ACTN1, etc
GO:0007045	BP	cell-substrate adherens junction assembly	0.010838	THY1; FN1
GO:0045216	BP	cell-cell junction organization	2.94E-06	DSG2; THY1; GJA1; FN1; WNT11; FSCN1
GO:0007043	BP	cell-cell junction assembly	0.028627	WNT11; FSCN1
GO:0034332	BP	adherens junction organization	0.048995	THY1; FN1
GO:0034329	BP	cell junction assembly	0.002273	THY1; FN1; WNT11; FSCN1
GO:0010811	BP	positive regulation of cell-substrate adhesion	0.00285	THY1; FN1; ABI3BP; EDIL3; FBLN1
GO:0034333	BP	adherens junction assembly	0.027114	THY1; FN1
GO:0005178	MF	integrin binding	2.56E-07	THY1; TGFBI; NOV; FN1; THBS4; EDIL3, etc
**Antioxidant related pathways**				
GO:0016209	MF	antioxidant activity	0.040595	MGST2; PTGS2; SOD3
GO:0004784	MF	superoxide dismutase activity	0.047928	SOD3
GO:0006801	BP	superoxide metabolic process	0.00047	NCF1; SOD3; CYBB
GO:1901031	BP	regulation of response to reactive oxygen species	0.048995	HGF
GO:0050664	MF	oxidoreductase activity, acting on NAD(P) H, oxygen as acceptor	0.01556	NCF1; CYBB
GO:0098869	BP	cellular oxidant detoxification	0.040595	MGST2; PTGS2; SOD3

In KEGG enrichment analysis, down-regulated genes in the H group were involved in ECM-receptor interaction (COL1A, THBS1, FN1, TN, ITGA5, ITGA8 and ITGB8), adherens junction (SHP-1, TGFβR, α-Actinin and Slug) and focal adhesion (Actinin and MLC) (Fig. S4, S5 and S6).

### Up-regulated genes in the COMB group

**Fig. 8:**
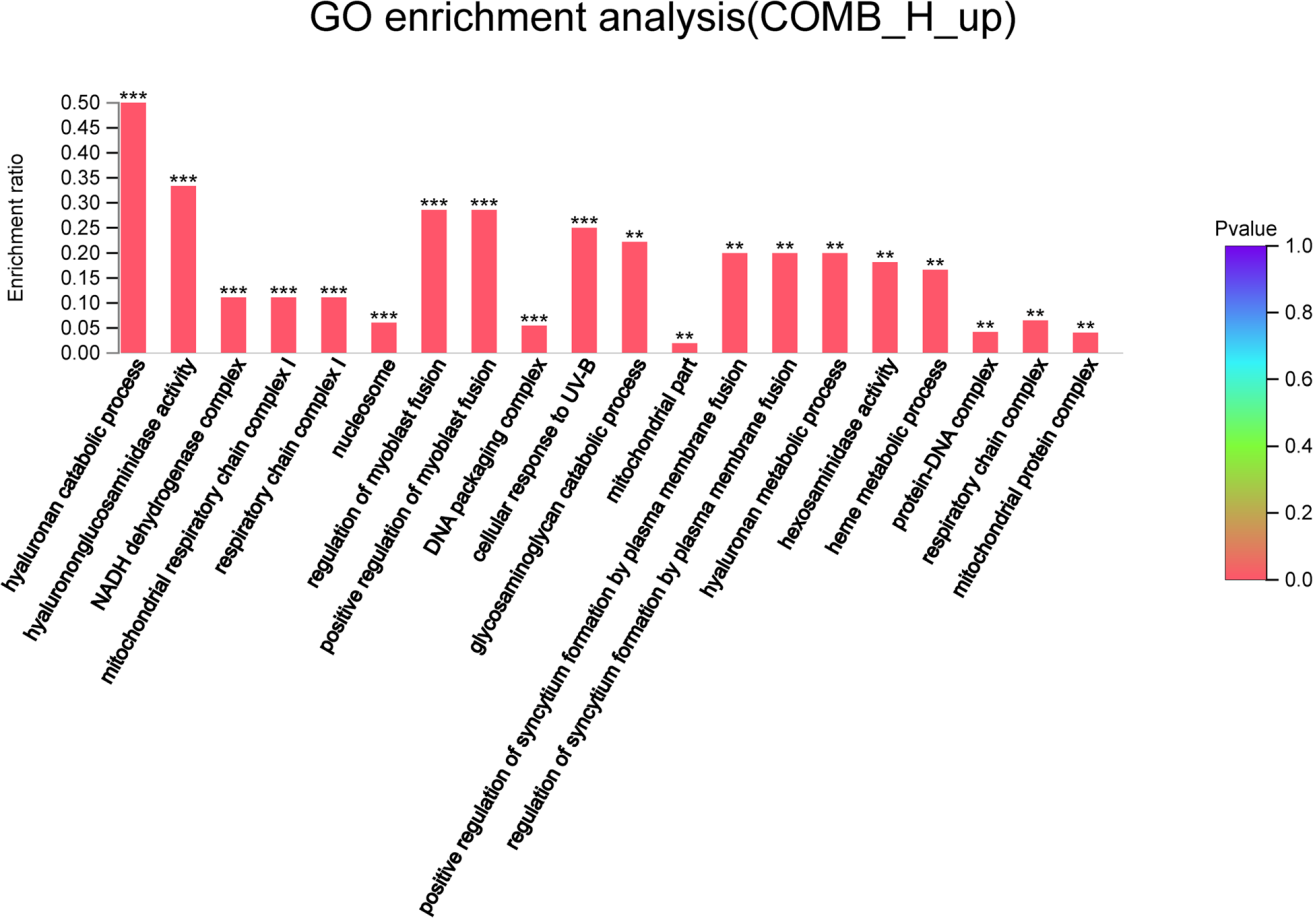
GO enrichment analysis of up-regulated genes in the COMB group. The abscissa indicates the GO term, and the ordinate indicates the enrichment ratio. “*“means *P* < 0.05, “**“means *P* < 0.01 and “***” means *P* < 0.001

Compared with those in the H group, up-regulated genes in the COMB group were involved in muscle development, hyaluronic acid synthesis, mitochondrial function, and redox pathway (Fig. 8).

The muscle development-related pathways enriched in the COMB group included positive regulation of muscle tissue development and muscle cell decision processes, which involved key genes such as MYF6, LMCD1 and TRPC3. Besides, the COMB group was enriched for mitochondria-associated pathways such as electron transport chains, mitochondrial respiratory chain complex I and mitochondrial protein complex pathways, which involved genes including TOMM6, NDUFV1, NDUFS5, NDUFB2, NDUFA2, LMCD1, ZNF593 and COASY (Table 10). The hyaluronic acid-related genes up-regulated in the COMB group included HYAL1 and HYAL3. Besides, the redox-related genes including LDHD, CPOX, SUOX, NDUFV1, GRHPR, DOHH and NDUFA2 were up-regulated in the COMB group, which were involved in the pathways such as redox process, NAD binding, NADPH binding and NADH dehydrogenase complex (Table 11). In KEGG enrichment analysis, up-regulated genes in the COMB group were involved in oxidative phosphorylation (NDUFS5, NDUFV1, NDUFA2, NDUFA13, NDUFB2, NDUFB7 and NDUFC2) (Fig. S7).

**Table 10:** Muscle development and mitochondria related pathway

Number	GO ID	Term Type	Description	***P-***value	Genes
**Muscle development related pathways**					
GO:1901741		BP	positive regulation of myoblast fusion	0.000717	MYF6
GO:0010831		BP	positive regulation of myotube differentiation	0.003478	MYF6
GO:0014743		BP	regulation of muscle hypertrophy	0.00447	LMCD1; TRPC3
GO:0048643		BP	positive regulation of skeletal muscle tissue development	0.00447	MYF6
GO:1901863		BP	positive regulation of muscle tissue development	0.020276	MYF6
GO:0051149		BP	positive regulation of muscle cell differentiation	0.025719	MYF6
GO:0048743		BP	positive regulation of skeletal muscle fiber development	0.035113	MYF6
GO:0045844		BP	positive regulation of striated muscle tissue development	0.017276	MYF6
GO:0051155		BP	positive regulation of striated muscle cell differentiation	0.011102	MYF6
GO:0048636		BP	positive regulation of muscle organ development	0.017276	MYF6
GO:0014744		BP	positive regulation of muscle adaptation	0.023546	TRPC3
**Mitochondria related pathways**					
GO:0042775		BP	mitochondrial ATP synthesis coupled electron transport	0.023546	NDUFV1
GO:0022904		BP	respiratory electron transport chain	0.025719	NDUFV1
GO:0022900		BP	electron transport chain	0.029237	NDUFV1
GO:0098803		CC	respiratory chain complex	0.002546	NDUFV1; NDUFS5; NDUFB2
GO:0045271		CC	respiratory chain complex I	0.000532	NDUFV1; NDUFS5; NDUFB2
GO:0005747		CC	mitochondrial respiratory chain complex I	0.000532	NDUFV1; NDUFS5; NDUFB2
GO:0098798		CC	mitochondrial protein complex	0.002727	TOMM6; NDUFV1; NDUFS5; NDUFB2
GO:0098800		CC	inner mitochondrial membrane protein complex	0.009305	NDUFV1; NDUFS5; NDUFB2
GO:0005742		CC	mitochondrial outer membrane translocase complex	0.046544	TOMM6
GO:0098779		BP	mitophagy in response to mitochondrial depolarization	0.04939	LMCD1; ZNF593

**Table 11:** Hyaluronan and redox related pathway

Number	GO ID	Term Type	Description	***P-***value	Genes
**Hyaluronan related pathways**					
GO:0030213		BP	hyaluronan biosynthetic process	0.023546	HYAL1
GO:0030214		BP	hyaluronan catabolic process	0.000207	HYAL3; HYAL1
GO:0030212		BP	hyaluronan metabolic process	0.001519	HYAL3; HYAL1
GO:1900106		BP	positive regulation of hyaluranon cable assembly	0.017711	HYAL1
GO:0004415		MF	hyalurononglucosaminidase activity	0.000514	HYAL3; HYAL1
GO:0033906		MF	hyaluronoglucuronidase activity	0.011842	HYAL3
GO:0036117		CC	hyaluranon cable	0.011842	HYAL1
GO:0050501		MF	hyaluronan synthase activity	0.017711	HYAL1
GO:0006027		BP	glycosaminoglycan catabolic process	0.00122	HYAL3; HYAL1
GO:0030203		BP	glycosaminoglycan metabolic process	0.039481	HYAL3; HYAL1
GO:0006026		BP	aminoglycan catabolic process	0.003025	HYAL3; HYAL1
GO:1903510		BP	mucopolysaccharide metabolic process	0.013602	HYAL3; HYAL1
**Redox related pathways**					
GO:0055114		BP	oxidation-reduction process	0.028445	LDHD; CPOX; SUOX; NDUFV1; GRHPR; DOHH; NDUFA2
GO:1990204		CC	oxidoreductase complex	0.006475	NDUFV1; NDUFS5; NDUFB2
GO:0016491		MF	oxidoreductase activity	0.045293	LDHD; CPOX; SUOX; NDUFV1; GRHPR; DOHH
GO:0016651		MF	oxidoreductase activity, acting on NAD(P)H	0.045042	NDUFV1
GO:0051287		MF	NAD binding	0.031684	NDUFV1; GRHPR
GO:0070402		MF	NADPH binding	0.040845	GRHPR
GO:0030964		CC	NADH dehydrogenase complex	0.000532	NDUFV1; NDUFS5; NDUFB2

### Down-regulated genes in the COMB group

**Fig. 9:**
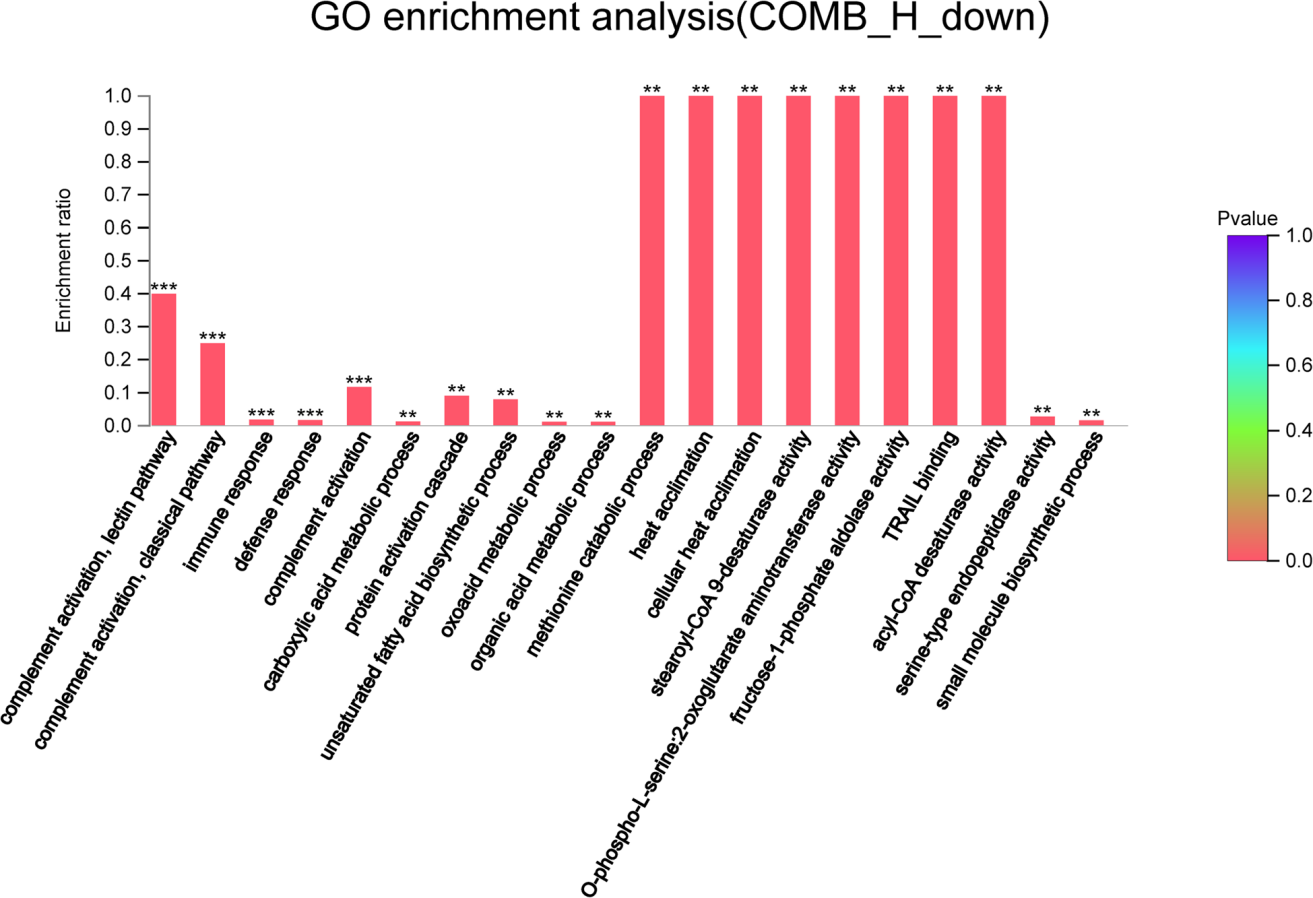
GO enrichment analysis of down-regulated genes in the COMB group. The abscissa indicates the GO term, and the ordinate indicates the enrichment ratio. “*“means *P* < 0.05, “**“means *P* < 0.01 and “***” means *P* < 0.001

Compared with those in the H group, down-regulated genes in the COMB group were involved in the inflammatory response, acid metabolism, fatty acid metabolism, and glycolysis-related pathways (Fig. 9).

The inflammatory response-related genes down-regulated in the COMB group included CCR5 and ALOX5 while the immune response-related genes included C1S, BLK, CCR5 and MARCH1 (Table 12). The acid metabolism-related pathways include organic acid synthesis process, oxoacid metabolism process and carboxylic acid synthesis process, which involved genes such as PSAT1, SCD, MAT1A, ALOX5, ST3GAL1 and ALDOB. The genes involved in fatty acid metabolism pathways include SCD and ALOX5. In addition, down-regulated genes in the COMB group were involved in glycolytic and carbohydrate metabolism, which included GALNT16, ST3GAL1, ALDOB and MAT1A (Table 13).

**Table 12:** Immune response and inflammatory response related pathways

Number	GO ID	Term Type	Description	***P-***value	Genes
**Immune response and inflammatory response related pathways**					
GO:0006954		BP	inflammatory response	0.004612	CCR5; ALOX5
GO:0002532		BP	production of molecular mediator involved in inflammatory response	0.01346	ALOX5
GO:0002538		BP	arachidonic acid metabolite production involved in inflammatory response	0.008097	ALOX5
GO:0002540		BP	leukotriene production involved in inflammatory response	0.008097	ALOX5
GO:0002253		BP	activation of immune response	0.005145	C1S; BLK
GO:0050778		BP	positive regulation of immune response	0.017271	C1S; BLK
GO:0045087		BP	innate immune response	0.005036	C1S; BLK
GO:0006956		BP	complement activation	0.000944	C1S
GO:0001867		BP	complement activation, lectin pathway	7.08E-05	C1S
GO:0006958		BP	complement activation, classical pathway	0.000197	C1S
GO:0004950		MF	chemokine receptor activity	0.047636	CCR5
GO:0016493		MF	C-C chemokine receptor activity	0.024101	CCR5
GO:0090026		BP	positive regulation of monocyte chemotaxis	0.024101	CCR5
GO:0002495		BP	antigen processing and presentation of peptide antigen via MHC class II	0.032008	MARCH1
GO:0042287		MF	MHC protein binding	0.045049	MARCH1

**Table 13:** Organic acid, faty acid metabolic process, glycolytic and carbohydrate metabolism related pathways

Number	GO ID	Term Type	Description	***P-***value	Genes
**Organic acid and faty acid metabolic process related pathways**					
GO:0006082		BP	organic acid metabolic process	0.002344	PSAT1; SCD; MAT1A; ALOX5; ST3GAL1; ALDOB
GO:0016053		BP	organic acid biosynthetic process	0.006961	PSAT1; SCD; ALOX5
GO:0043436		BP	oxoacid metabolic process	0.002254	PSAT1; SCD; MAT1A; ALOX5; ST3GAL1; ALDOB
GO:0046394		BP	carboxylic acid biosynthetic process	0.006961	PSAT1; SCD; ALOX5
GO:0019752		BP	carboxylic acid metabolic process	0.001555	PSAT1; SCD; MAT1A; ALOX5; ST3GAL1; ALDOB
GO:0006633		BP	fatty acid biosynthetic process	0.012538	SCD; ALOX5
GO:0006636		BP	unsaturated fatty acid biosynthetic process	0.002054	SCD; ALOX5
GO:0016215		MF	acyl-CoA desaturase activity	0.002706	SCD
**Glycolytic and carbohydrate metabolism related pathways**					
GO:0030388		BP	fructose 1,6-bisphosphate metabolic process	0.01613	ALDOB
GO:0006000		BP	fructose metabolic process	0.018794	ALDOB
GO:0070061		MF	fructose binding	0.010782	ALDOB
GO:0061609		MF	fructose-1-phosphate aldolase activity	0.002706	ALDOB
GO:0004332		MF	fructose-bisphosphate aldolase activity	0.010782	ALDOB
GO:0005975		BP	carbohydrate metabolic process	0.029095	GALNT16; ST3GAL1; ALDOB
GO:0030246		MF	carbohydrate binding	0.041651	GALNT16; ALDOB

In KEGG enrichment analysis, genes involved in the regulation of lipolysis in adipocytes (PLIN), glycolysis/gluconeogenesis (ALDO) and arachidonic acid metabolism (ALOX5) were down-regulated in the COMB group (Fig. S8, S9 and S10).

### Transcriptome differential gene verification

**Fig. 10:**
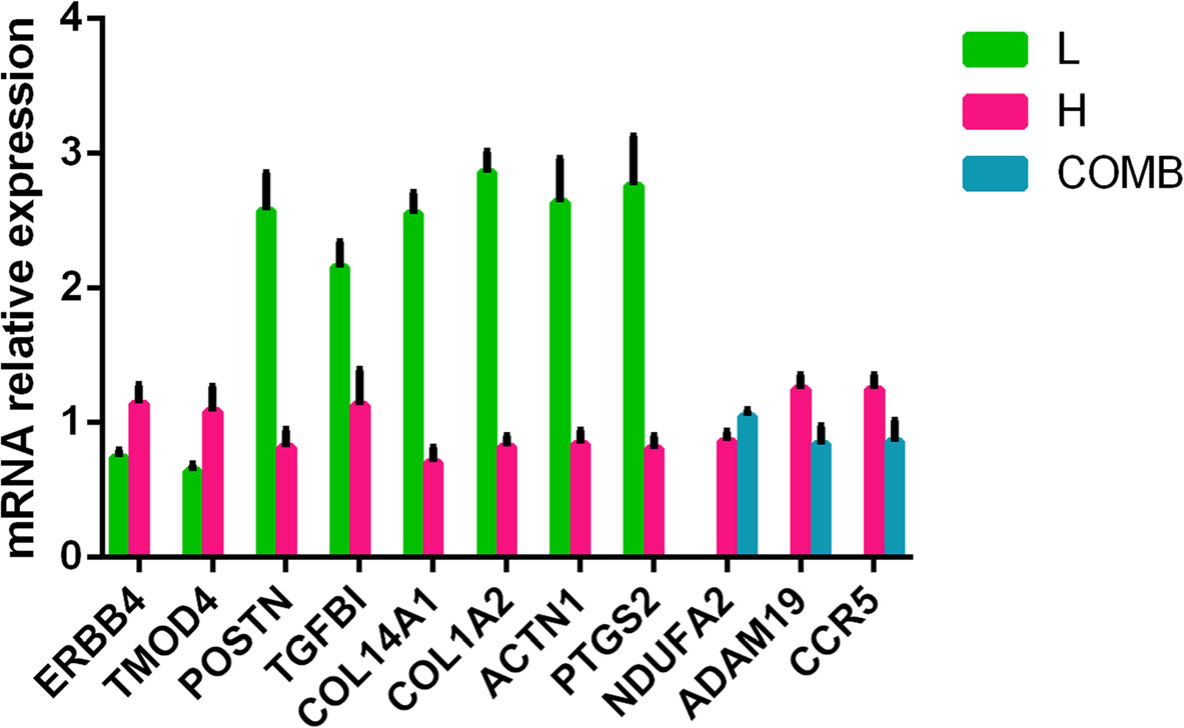
The mRNA relative expression of DEGs quantified by quantitative reverse transcription-PCR. Data presented as means ± SEM

The transcriptome differential genes were verified by real-time PCR, and the gene expression pattern was consistent with the transcriptome results (Fig. 10).

## Discussion

In the current study, the H group showed significantly increased cooking loss of breast muscle when compared with the L group. The muscle disease such as PSE (Pale, Soft and Exudative) meat [30] and wooden breast [31] have higher cooking loss than normal meat.

Stress is an essential cause of the decline in meat quality. In this study, the activity of LDH in the H group was higher than that in the L group. In transcriptome analysis, the enriched genes in the H group were involved in stimuli response pathway. In the H group, genes encoding nitric oxide synthase 1 (NOS1), Kelch-Like ECH-associated protein 1 (KEAP1) and cyclin-dependent kinase inhibitor 1A (p21, Cip1) (CDKN1A) were up-regulated. High levels of NO reduce the antioxidant capacity of post-mortem muscles, increasing the accumulation of ROS and reactive nitrogen, resulting in high levels of protein oxidation. Studies have shown that inhibition of nitric oxide synthase can significantly reduce protein carbonyl content and protein oxidation [32]. Inhibition of CDKN1A expression by miRNAs promotes myoblast proliferation [33]. Up-regulation of KEAP1 expression increases the degradation of Nrf2 in cells, making cells more susceptible to free radical damage [34]. Heat stress can reduce the oxidative stability of broiler muscle protein and reduce the strength of the myofibrillar gel, resulting in increased drip loss and cooking loss in broilers [35]. A study has shown that genes involved in the stimulation response pathway are significantly enriched in muscles with high drip loss [36]. Therefore, increased expression of stress pathway-related genes such as KEAP1 and CDKN1A may be one of the causes of muscle quality deterioration.

This study found that the H group had the fastest pH decline rate. The rapid decline in pH is usually accompanied by an increase in the rate of glycolysis and the accumulation of lactic acid, resulting in a decrease of muscle function [37]. In this study, high stocking density led to up-regulation of genes involved in glycolysis and fat metabolism pathways. Anaerobic glycolysis is a vital energy metabolism pathway for post-mortem broilers. Under anaerobic conditions, muscle glycogen degradation occurs through glycolysis, which causes pyruvate to synthesize lactic acid, thus leading to a decrease in muscle pH due to the accumulation of lactic acid [38, 39]. High stocking density in this study also caused up-regulation of striated muscle contraction pathway-related genes such as SIX homeobox 1 (Six1). It has been found that white streak muscles have up-regulated expression of striated muscle contraction-related genes compared with normal meat [40]. Six1 converts slow muscle fibres into fast muscle fibres [41, 42]. The proportion of fast muscle fibres was negatively correlated with post-mortem pH [43]. Besides, the enriched genes in the H group were involved in calcium transport, sodium transport, and cation transport. Importantly, ion balance is the basis for maintaining normal physiological functions. Abnormal metabolism caused by high concentrations of calcium ions may be associated with the incidence of turkey PSE [44]. Furthermore, changes in muscle cation homeostasis may mark the beginning of muscle degeneration [45] and cause a reduction in meat quality [46].

Dietary supplementation with niacin (nicotinamide precursor) at 60 mg/kg was reported to reduce the drip loss of breast muscles in broilers [14]. In our study, the COMB group showed significantly reduced drip loss and cooking loss compared with the H group. Further, the COMB group showed significantly decreased activity of LDH compared to the H group. Besides, the COMB group showed inhibited expression of glycolytic and inflammation genes [37].

In KEGG enrichment analysis, the enriched genes in the H group were involved in inflammatory mediator regulation of RTP channels and chemokine signalling pathway. In contrast, the up-regulated genes in the COMB group were involved in the inflammatory response. Macrophage infiltration in the pectoral muscle might cause muscle damage [47]. The muscle disease such as white striped muscle is usually accompanied by elevated expression of immune-related genes [40]. During tissue degeneration, immune cells immediately enter the site of injury, triggering an inflammatory response, and attracting more immune cells to the damaged area. It can cause phagocytosis of cell debris and release of cytokines, prostaglandins and other signalling proteins, resulting in interstitial spaces [48].

We found that key genes down-regulated in the H group, such as MYOZ2, were involved in muscle development, cell adhesion, cell matrix, collagen, and cytoskeleton. MYOZ2 belongs to sarcomeric family and links calcineurin to alpha-actinin at the Z-line of skeletal muscle sarcomere and can play a role in skeletal muscle differentiation and growth [49]. It was suggested that MYOZ2 knockout mice had neuromuscular disease [50]. Also, genes down-regulated in the H group were involved in cell matrix and collagen pathways. Extracellular matrix (ECM) is a major macromolecule in skeletal muscle and has a substantial effect on meat quality. The remodelling of ECM is mainly regulated by matrix metalloproteinases. The expression of matrix metalloproteinase-1 is negatively correlated with cooking loss and positively correlated with hydraulic performance [51]. Collagen is an abundant connective tissue protein that is an important factor in the tenderness and texture of the meat and is well resistant to physical damage during cooking [52]. The addition of collagen increases the ability of pork [53] and poultry [54] to combine with water and reduces cooking losses. Furthermore, high stocking density downregulates cell adhesion, cytoskeletal and integrin binding-related genes such as integrin subunit alpha 8 (ITGA8), integrin subunit beta 8 (ITGB8) and integrin subunit beta like 1 (ITGBL1). Proteolytic degradation of cell adhesion proteins is associated with the production of drip channels [55]. The cytoskeleton is a highly complex network composed of a large number of connections between myofibrils and myofibrillar membranes. Degradation of the cytoskeleton causes extracellular water to flow into the muscle cells, thereby increasing drip loss [56]. Integrins are heterodimeric cell adhesion molecules that bind the extracellular matrix to the cytoskeleton and play an essential role in controlling cell membrane-cytoskeletal attachment and signalling pathways [57]. The β-chain integrin is responsible for the attachment of the cell membrane to the cytoskeleton [58]. Degradation of β1 integrin promotes the formation of water channels between cells and cell membranes, thereby increasing drip loss [59]. In addition, it has been found that integrins are inversely related to pork drip loss [60].

Compared with the H group, the COMB group showed up-regulation of muscle development, hyaluronic acid levels, mitochondrial function, and the redox pathway. Studies have found that hyaluronic acid is a crucial water-holding molecule [61, 62]. Furthermore, supplementation with antioxidant isoflavones can be achieved by reducing lipid peroxidation and increasing oxidative stability in the pectoral muscles [63]. Therefore, enhanced hyaluronic acid biosynthesis and antioxidant capacity may improve muscle quality.

Additionally, up-regulated genes in the COMB group involved the complex I-related gene NDUFS5. The mitochondrial respiratory chain (MRC) consists of four membrane-bound electron transport protein complexes (I-IV) and ATP synthase (complex V) that produce ATP for cellular processes. Complex I deficiency, NADH ubiquinone oxidoreductase is the most common form of MRC dysfunction and is associated with a variety of diseases [64, 65]. Complex I deficiency leads to various physiological disorders such as ATP depletion, calcium homeostasis, ROS accumulation [66] and induction of apoptosis [67]. A study found that mitochondrial and oxidative phosphorylation-related gene expression was negatively correlated with drip loss. A negative correlation with drip loss means that there is a decrease in the number of mitochondria in muscles with high drip loss [68].

## Conclusion

**Fig. 11:**
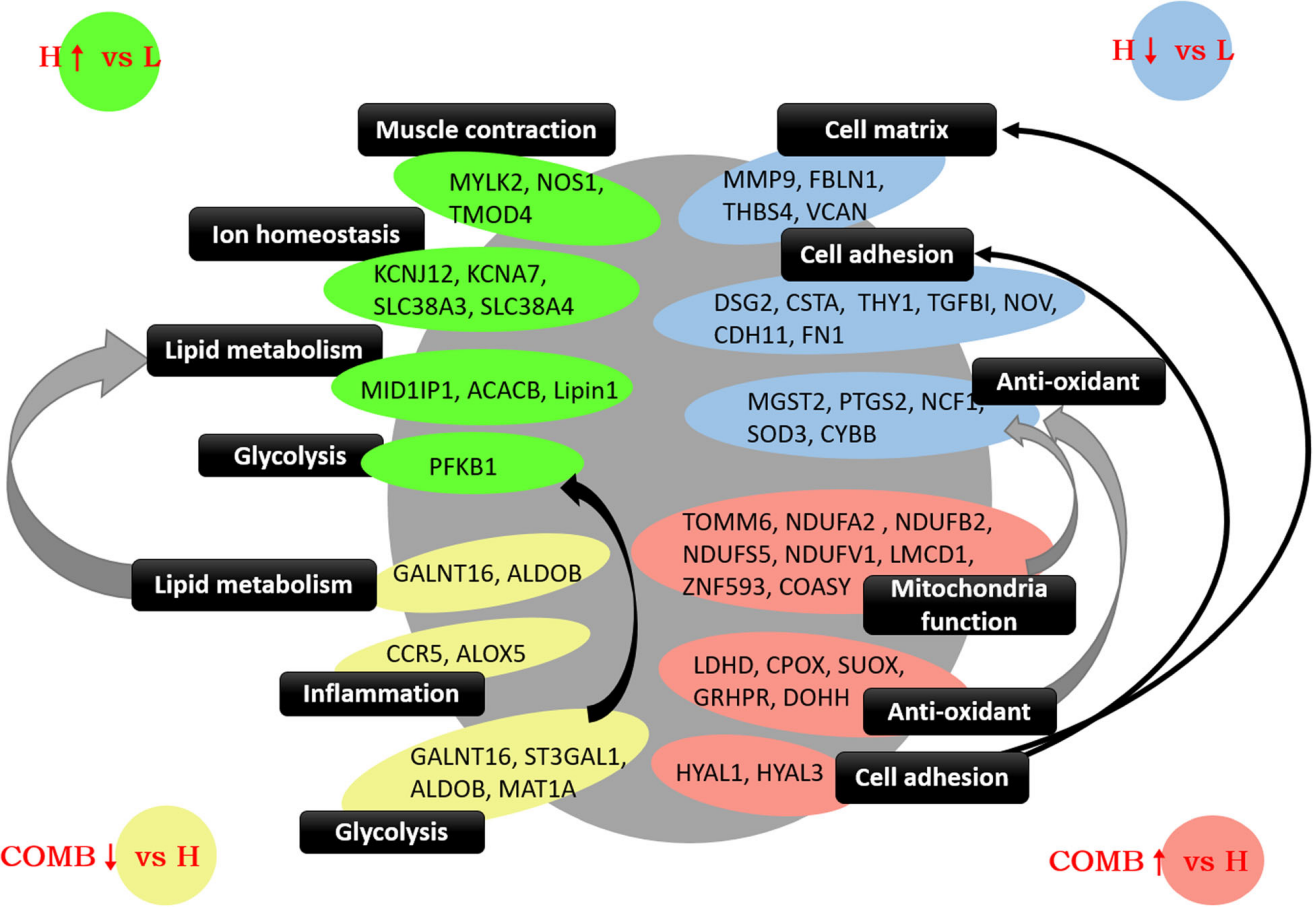
The graphic description of the normalization effect of nicotinamide and sodium butyrate on breast muscle. This is the original graph drafted by the authors of this article

High stocking density may cause oxidative stress, abnormal muscle contraction, and abnormal metabolism of glycolipids; destroy ion channels and cell matrix; reduce muscle strength by inhibiting muscle development, and cell adhesion and collagen synthesis, all of which result in reduced muscle function. Supplementation with NAM and BA in combination can improve mitochondrial function and antioxidant capacity, and inhibit inflammatory response and glycolysis by promoting muscle development and hyaluronic acid synthesis, thereby reducing drip loss of the breast muscle and improving muscle quality (Fig. 11).

## Methods

### Experimental birds, diets, and management

Amount of 300 Cobb broilers (21-day-old) were divided into 3 groups: low stocking density (L, 14 birds/m^2^), high stocking density (H, 18 birds/m^2^) and combination of NAM and BA (COMB, 18 birds/m^2^), with 6 replicates for each group. The stocking densities of this study are referred to Vargas-Galicia et al. [69]. The L and H groups were fed a basal diet. The COMB group was fed basal diet supplemented with 50 mg/kg NAM and 500 mg/kg BA. The dosage 50 mg/kg NAM and 500 mg/kg BA used in this study were based on our previous studies [70, 71]. Experimental diets were designed to meet nutrient requirements of National Research Council (1994) [72]. The nutrient levels and composition of basic diet were shown in Table 14. Broilers in this study were raised from 21-day-old to 42-day-old, and feed and water were provided ad libitum.

**Table 14:** The composition and nutrient level of basal diet

Ingredient	Percent	Nutrients	Percent
Corn	62.05	Metabolic energy	3100 Kcal
Soybean meal	26.90	Crude Protein	18.98
Corn Gluten Meal	4.00	Lysine	1.04
Soybean oil	3.10	Methionine	0.49
DL-Methionine	0.18	Threonine	0.74
L-Lysine sulphate	0.40	Tryptophan	0.24
Sodium chloride	0.30	Calcium	0.86
Choline chloride (50%)	0.15	Available Phosphorus	0.32
Vitamin premix^b^	0.02	Met+Cys^c^	0.80
Trace mineral premix^a^	0.20		
Dicalcium phosphate	1.40		
Limestone	1.20		
Phytase	0.02		
Antioxidant	0.03		
Medical stone	0.05		

^a^ The trace mineral premix provided the following per kg of diets: Cu, 16 mg (as CuSO_4_·5H_2_O); Zn, 110 mg (as ZnSO_4_); Fe, 80 mg (as FeSO_4_·H_2_O); Mn, 120 mg (as MnO); Se, 0.3 mg (as Na_2_SeO_3_); I, 1.5 mg (as KI); Co, 0.5 mg

^b^ The vitamin premix provided the following per kg of diets: vitamin A, 10,000 IU; vitamin D3, 2400 IU; vitamin E, 20 mg; vitamin K3, 2 mg; vitamin B1, 2 mg; vitamin B2, 6.4 mg; VB6, 3 mg; VB12, 0.02 mg; biotin, 0.1 mg; folic acid, 1 mg; pantothenic acid, 10 mg; nicotinamide, 30 mg

^c^ Met+Cys: Methionine+ Cysteine

### Production performance determination and sample collection

On 42-day, remove feed for 5 h and record the remaining feed per cage, then weight the body weight (BW) of broilers. Calculate body weight gain (BWG), feed intake (FI) and the feed conversion rate (FCR).

For breast collection, one broiler per replicate was randomly selected and euthanized by intravenous injection of pentobarbital sodium (390 mg/ml) at a dose of 300 mg/kg. The breast muscle was collected for meat quality analysis and further study.

Each group had six replicates for the determination of meat quality, enzyme activities and mRNA relative expression; there were three biology replicates in each group for RNA-sequencing.

### Meat quality analysis

The meat quality of right side major pectoral muscle was quickly determined after slaughtering. The drip loss was determined according to Liu et al. [73]. Cooking loss was measured according to the protocol described by Cai et al. [74]. The pH values of the pectoral muscle at 45 min and 24 h were measured by a pH meter (testo 205; Germany). Each sample was tested at 3 different locations (top, middle and bottom) and the average of 3 measurements was calculated.

### Enzyme activity determination in breast muscle

The total antioxidant capacity (T-AOC, cat#A015), anti-superoxide anion (cat#A052), the activities of creatine kinase (CK, cat#A032), lactate dehydrogenase (LDH, cat#A020–2), malic dehydrogenase (MDH, cat#A021–2), and the content of hydroxyproline (cat# A030–2) in breast muscle were measured by commercial analytical kits (Jian Cheng Bioengineering Institute, Nanjing, China).

### RNA extraction, library preparation and Illumina Hiseq X ten sequencing

Total RNA from the breast muscle was extracted by TRIzol® Reagent (Invitrogen, Carlsbad, CA, USA). The RNA quality was then measured by 2100 Bioanalyser (Agilent Technologies, Santa Clara, CA, USA) and quantified using the ND-2000 (Nanodrop Technologies, Wilmington, Delaware).

RNA-seq library was constructed according to TruSeqTM RNA sample preparation Kit from Illumina (San Diego, CA, USA), then was sequenced with the Illumina HiSeq X Ten (2 × 150 bp read length).

### Read mapping, differential expression analysis and functional enrichment

SeqPrep and Sickle were applied to process raw paired-end reads. Then use TopHat version2.0.0 [75] software to align the clean reads to the reference genome.

FRKM method was applied to identify differentially expressed genes (DEGs). RSEM [76] was used to quantify gene abundances. Differential gene expression was analyzed by R statistical package software EdgeR [77]. Goatools and KOBAS [78] were applied for KEGG pathway enrichment and GO functional analysis.

### The mRNA expression of muscle developmental genes

Several differentially expressed genes involved muscle development were validated by real-time PCR analysis. The mRNA expression of muscle was determined as we previously described [71]. The primer sequences of target gene and housekeeping gene beta-actin were shown in Table 15. The results of gene expression were analyzed and compared using 2^-ΔΔCT^.

**Table 15:** Real time PCR primer sequence

Gene	Primer sequence (5′-3′)	Size	Accession NO.
*GAPDH*	Forward: GGTAGTGAAGGCTGCTGCTGATG	200	NM_204305.1
	Reverse: AGTCCACAACACGGTTGCTGTATC		
*ERBB4*	Forward: ATCACCAGCATCGAGCACAACAG	114	NM_001030365.1
	Reverse: CAGGTTCTCCAGTGGCAGGTATTC		
*TMOD4*	Forward: GATGGAGATGGCGACGATGCTG	135	NM_204774.1
	Reverse: TTCTTCTGCTTGCGACGGAGTTC		
*PTGS2*	Forward: ACTGCTGGCCGCTCTCCTTG	121	NM_001167719.1
	Reverse: CCTCGTGCAGTCACATTCATACCG		
*COL1A2*	Forward: TCCTCCTGGTAACAACGGTCCTG	85	NM_001079714.2
	Reverse: GAGACCATTGCGACCATCCTTACC		
*POSTN*	Forward: CAGCCGCATCTGCTCACTATGAC	200	NM_001030541.1
	Reverse: CTTCATGTAGCCAGGACAGCACTC		
*COL14A1*	Forward: CCAACTCAGCCACCAACTTCTCC	107	NM_205334.1
	Reverse: TCCACTAGGAACACCAGGTCAGC		
*TGFBI*	Forward: ACCACCACGAACAGCATTCAGC	87	NM_205036.1
	Reverse: GTTGAGGTCAGAAGCAGCCACAG		
*ACTN1*	Forward: GCGTGGAACAGATTGCTGCTATTG	88	NM_204127.1
	Reverse: ATCTTCTGGCACCTGGCATTGAC		
*NDUFA2*	Forward: CATCGAGCAGCACTACGTGACTC	159	NM_001302137.1
	Reverse: TTGGCAACTTCATCCACACTGAGG		
*ADAM19*	Forward: GACAGGACAAGCACGGACCATC	166	NM_001195122.1
	Reverse: AGGAAGCGGCTCCAGGACATAG		
*CCR5*	Forward: GAGATGCGCTGTGCCGGATTC	159	NM_001271141.1
	Reverse: TGCTGGTGAGGATGCCGTAGG		

### Statistical analysis

The results are expressed as means with their standard error mean (SEM). SPSS 20.0 for Windows (SPSS Inc. Chicago, IL) was applied for One-way ANOVA analysis. Significant difference was considered at *P* < 0.05.

## Supplementary information

**Additional file 1: Figure S1.** Inflammatory mediator regulation of RTP channels pathway analysis. Differential expressed genes that are involved in the inflammatory mediator regulation of RTP channels [map 04750], are highlighted. We thank Kanehisa Laboratories for providing the copyright permission of KEGG pathway maps [29].

**Additional file 2: Figure S2.** Chemokine signaling pathway analysis. Differential expressed genes that are involved in the chemokine signaling pathway [map 04062], are highlighted. We thank Kanehisa Laboratories for providing the copyright permission of KEGG pathway maps [29].

**Additional file 3: Figure S3.** Calcium signaling pathway analysis. Differential expressed genes that are involved in the calcium signaling pathway [map 04020], are highlighted. We thank Kanehisa Laboratories for providing the copyright permission of KEGG pathway maps [29].

**Additional file 4: Figure S4.** ECM-receptor interaction pathway analysis. Differential expressed genes that are involved in the inflammatory mediator regulation of RTP channels [map 04512], are highlighted. We thank Kanehisa Laboratories for providing the copyright permission of KEGG pathway maps [29].

**Additional file 5: Figure S5.** Adherens junction pathway analysis. Differential expressed genes that are involved in the adherens junction [map 04520], are highlighted. We thank Kanehisa Laboratories for providing the copyright permission of KEGG pathway maps [29].

**Additional file 6: Figure S6.** Focal adhesion pathway analysis. Differential expressed genes that are involved in the focal adhesion [map 04510], are highlighted. We thank Kanehisa Laboratories for providing the copyright permission of KEGG pathway maps [29].

**Additional file 7: Fig. S7.** Oxidative phosphorylation pathway analysis. Differential expressed genes that are involved in the oxidative phosphorylation [map 00190], are highlighted. We thank Kanehisa Laboratories for providing the copyright permission of KEGG pathway maps [29].

**Additional file 8: Figure S8.** Regulation of lipolysis in adipocytes pathway analysis. Differential expressed genes that are involved in the Regulation of lipolysis in adipocytes [map 04923], are highlighted. We thank Kanehisa Laboratories for providing the copyright permission of KEGG pathway maps [29].

**Additional file 9: Figure S9.** Glycolysis/Gluconeogenesis pathway analysis. Differential expressed genes that are involved in the Glycolysis/Gluconeogenesis [map 00010], are highlighted. We thank Kanehisa Laboratories for providing the copyright permission of KEGG pathway maps [29].

**Additional file 10: Figure S10.** Arachidonic acid metabolism pathway analysis. Differential expressed genes that are involved in the arachidonic acid metabolism [map 00590], are highlighted. We thank Kanehisa Laboratories for providing the copyright permission of KEGG pathway maps [29].

## Data Availability

All the sequencing data are deposited in SRA under the Bioproject accession number PRJNA558637.

## Abbreviations

L: Low stocking density group
H: High stocking density group
COMB: A combination of nicotinamide and sodium butyrate group
NAM: Nicotinamide
ROS: Reactive oxygen species
BA: Butyrate sodium
PGC1α: Peroxisome proliferator-activated receptor-γ coactivator 1α
NAD: Nicotinamide adenine dinucleotide
T-AOC: Total antioxidant capacity
LDH: Lactate dehydrogenase
CK: Creatine kinase
MDH: Malic dehydrogenase
DEGs: Differentially expressed genes
NOS1: Nitric oxide synthase 1
KEAP1: Kelch-Like ECH-associated protein 1
CDKN1A: Cyclin-dependent kinase inhibitor 1A

## References

[1] Najafi P, Zulkifli I, Jajuli NA, Farjam AS, Ramiah SK, Amir AA, O'Reily E, Eckersall D (2015). Environmental temperature and stocking density effects on acute phase proteins, heat shock protein 70, circulating corticosterone and performance in broiler chickens. Int J Biometeorol.

[2] Zhang YR, Zhang LS, Wang Z, Liu Y, Li FH, Yuan JM, Xia ZF (2018). Effects of stocking density on growth performance, meat quality and tibia development of Pekin ducks. Anim Sci J.

[3] Patria C, Afnan R, Arief II (2016). Physical and microbiological qualities of kampong-broiler crossbred chickens meat raised in different stocking densities. Media Peternakan.

[4] Falowo AB, Fayemi PO, Muchenje V (2014). Natural antioxidants against lipid-protein oxidative deterioration in meat and meat products: a review. Food Res Int.

[5] Zhang L, Yue HY, Wu SG, Xu L, Zhang HJ, Yan HJ, Cao YL, Gong YS, Qi GH (2010). Transport stress in broilers. II. Superoxide production, adenosine phosphate concentrations, and mRNA levels of avian uncoupling protein, avian adenine nucleotide translocator, and avian peroxisome proliferator-activated receptor-gamma coactivator-1alpha in skeletal muscles. Poult Sci.

[6] Selman C, McLaren JS, Himanka MJ, Speakman JR (2000). Effect of long-term cold exposure on antioxidant enzyme activities in a small mammal. Free Radic Biol Med.

[7] Zhang C, Yang L, Zhao X, Chen X, Wang L, Geng Z (2018). Effect of dietary resveratrol supplementation on meat quality, muscle antioxidative capacity and mitochondrial biogenesis of broilers. J Sci Food Agric.

[8] Choi HJ, Jang SY, Hwang ES (2015). High-dose Nicotinamide suppresses ROS generation and augments population expansion during CD8(+) T cell activation. Mol Cells.

[9] Kwak JY, Ham HJ, Kim CM, Hwang ES (2015). Nicotinamide exerts antioxidative effects on senescent cells. Mol Cells.

[10] Mitchell SJ, Bernier M, Aon MA, Cortassa S, Kim EY, Fang EF, Palacios HH, Ali A, Navas-Enamorado I, Di Francesco A (2018). Nicotinamide Improves Aspects of Healthspan, but Not Lifespan, in Mice. Cell Metab.

[11] Jahns F, Wilhelm A, Jablonowski N, Mothes H, Greulich KO, Glei M (2015). Butyrate modulates antioxidant enzyme expression in malignant and non-malignant human colon tissues. Mol Carcinog.

[12] Zhang WH, Jiang Y, Zhu QF, Gao F, Dai SF, Chen J, Zhou GH (2011). Sodium butyrate maintains growth performance by regulating the immune response in broiler chickens. Brit Poultry Sci.

[13] Walsh ME, Bhattacharya A, Sataranatarajan K, Qaisar R, Sloane L, Rahman MM, Kinter M, Van Remmen H (2015). The histone deacetylase inhibitor butyrate improves metabolism and reduces muscle atrophy during aging. Aging Cell.

[14] Jiang RR, Zhao GP, Chen JL, Zheng MQ, Zhao JP, Li P, Hu J, Wen J (2011). Effect of dietary supplemental nicotinic acid on growth performance, carcass characteristics and meat quality in three genotypes of chicken. J Anim Physiol Anim Nutr (Berl).

[15] Panda A, Rao S, Raju M, Sunder GS (2009). Effect of butyric acid on performance, gastrointestinal tract health and carcass characteristics in broiler chickens. Asian-Australas J Anim Sci.

[16] Xiong J, Qiu H, Bi Y, Zhou H, Guo S, Ding B (2018). Effects of dietary supplementation with Tributyrin and coated sodium butyrate on intestinal morphology, Disaccharidase activity and intramuscular fat of lipopolysaccharide-challenged broilers. Braz J Poult Sci.

[17] Wenz T, Rossi SG, Rotundo RL, Spiegelman BM, Moraes CT (2009). Increased muscle PGC-1alpha expression protects from sarcopenia and metabolic disease during aging. Proc Natl Acad Sci U S A.

[18] Mouchiroud L, Houtkooper RH, Moullan N, Katsyuba E, Ryu D, Canto C, Mottis A, Jo YS, Viswanathan M, Schoonjans K (2013). The NAD(+)/Sirtuin pathway modulates longevity through activation of mitochondrial UPR and FOXO signaling. Cell.

[19] Zhang H, Ryu D, Wu Y, Gariani K, Wang X, Luan P, D'Amico D, Ropelle ER, Lutolf MP, Aebersold R (2016). NAD(+) repletion improves mitochondrial and stem cell function and enhances life span in mice. Science.

[20] Hathorn T, Snyder-Keller A, Messer A (2011). Nicotinamide improves motor deficits and upregulates PGC-1alpha and BDNF gene expression in a mouse model of Huntington's disease. Neurobiol Dis.

[21] Frederick DW, Loro E, Liu L, Davila A, Chellappa K, Silverman IM, Quinn WJ, Gosai SJ, Tichy ED, Davis JG (2016). Loss of NAD homeostasis leads to progressive and reversible degeneration of skeletal muscle. Cell Metab.

[22] Goody MF, Kelly MW, Reynolds CJ, Khalil A, Crawford BD, Henry CA (2012). NAD+ biosynthesis ameliorates a zebrafish model of muscular dystrophy. PLoS Biol.

[23] Vrablik TL, Wang W, Upadhyay A, Hanna-Rose W (2011). Muscle type-specific responses to NAD+ salvage biosynthesis promote muscle function in Caenorhabditis elegans. Dev Biol.

[24] Khan M, Ringseis R, Mooren FC, Kruger K, Most E, Eder K (2013). Niacin supplementation increases the number of oxidative type I fibers in skeletal muscle of growing pigs. BMC Vet Res.

[25] Khan M, Couturier A, Kubens JF, Most E, Mooren FC, Kruger K, Ringseis R, Eder K (2013). Niacin supplementation induces type II to type I muscle fiber transition in skeletal muscle of sheep. Acta Vet Scand.

[26] Gao Z, Yin J, Zhang J, Ward RE, Martin RJ, Lefevre M, Cefalu WT, Ye J (2009). Butyrate improves insulin sensitivity and increases energy expenditure in mice. Diabetes.

[27] Huang Y, Gao S, Jun G, Zhao R, Yang X (2017). Supplementing the maternal diet of rats with butyrate enhances mitochondrial biogenesis in the skeletal muscles of weaned offspring. Br J Nutr.

[28] Henagan TM, Stefanska B, Fang Z, Navard AM, Ye J, Lenard NR, Devarshi PP (2015). Sodium butyrate epigenetically modulates high-fat diet-induced skeletal muscle mitochondrial adaptation, obesity and insulin resistance through nucleosome positioning. Br J Pharmacol.

[29] Kanehisa M, Goto S (2000). KEGG: Kyoto encyclopedia of genes and genomes. Nucleic Acids Res.

[30] Van Laack RL, Liu CH, Smith MO, Loveday HD (2000). Characteristics of pale, soft, exudative broiler breast meat. Poult Sci.

[31] Mudalal S, Lorenzi M, Soglia F, Cavani C, Petracci M (2015). Implications of white striping and wooden breast abnormalities on quality traits of raw and marinated chicken meat. Animal.

[32] Zhang W, Marwan AH, Samaraweera H, Lee EJ, Ahn DU (2013). Breast meat quality of broiler chickens can be affected by managing the level of nitric oxide. Poult Sci.

[33] Wang J, Song C, Cao X, Li H, Cai H, Ma Y, Huang Y, Lan X, Lei C, Ma Y (2019). MiR-208b regulates cell cycle and promotes skeletal muscle cell proliferation by targeting CDKN1A. J Cell Physiol.

[34] Kensler TW, Wakabayashi N, Biswal S (2007). Cell survival responses to environmental stresses via the Keap1-Nrf2-ARE pathway. Annu Rev Pharmacol Toxicol.

[35] Wang RR, Pan XJ, Peng ZQ (2009). Effects of heat exposure on muscle oxidation and protein functionalities of pectoralis majors in broilers. Poult Sci.

[36] Wimmers K, Murani E, Ponsuksili S (2010). Functional genomics and genetical genomics approaches towards elucidating networks of genes affecting meat performance in pigs. Brief Funct Genomics.

[37] Huang JC, Yang J, Huang M, Zhu ZS, Sun XB, Zhang BH, Xu XL, Meng WG, Chen KJ, Xu BC (2018). Effect of pre-slaughter shackling and wing flapping on plasma parameters, postmortem metabolism, AMPK, and meat quality of broilers. Poult Sci.

[38] Zeferino CP, Komiyama CM, Pelicia VC, Fascina VB, Aoyagi MM, Coutinho LL, Sartori JR, Moura AS (2016). Carcass and meat quality traits of chickens fed diets concurrently supplemented with vitamins C and E under constant heat stress. Animal.

[39] Huang JC, Yang J, Huang F, Huang M, Chen KJ, Xu XL, Zhou GH (2016). Effect of fast pH decline during the early postmortem period on calpain activity and cytoskeletal protein degradation of broiler M. pectoralis major. Poult Sci.

[40] Marchesi J, Ibelli A, Peixoto JO, Cantao ME, Pandolfi J, Marciano C, Zanella R, Settles ML, Coutinho LL, Ledur MC (2019). Whole transcriptome analysis of the pectoralis major muscle reveals molecular mechanisms involved with white striping in broiler chickens. Poult Sci.

[41] Wu W, Huang R, Wu Q, Li P, Chen J, Li B, Liu H (2014). The role of Six1 in the genesis of muscle cell and skeletal muscle development. Int J Biol Sci.

[42] Sakakibara I, Wurmser M, Dos Santos M, Santolini M, Ducommun S, Davaze R, Guernec A, Sakamoto K, Maire P (2016). Six1 homeoprotein drives myofiber type IIA specialization in soleus muscle. Skelet Muscle.

[43] Ryu YC, Lee MH, Lee SK, Kim BC (2006). Effects of muscle mass and fiber type composition of longissimus dorsi muscle on postmortem metabolic rate and meat quality in pigs. J Muscle Foods.

[44] Strasburg GM, Chiang W (2009). Pale, soft, exudative Turkey--the role of ryanodine receptor variation in meat quality. Poult Sci.

[45] Sandercock DA, Mitchell MA (2004). The role of sodium ions in the pathogenesis of skeletal muscle damage in broiler chickens. Poult Sci.

[46] Sandercock DA, Barker ZE, Mitchell MA, Hocking PM (2009). Changes in muscle cell cation regulation and meat quality traits are associated with genetic selection for high body weight and meat yield in broiler chickens. Genet Sel Evol.

[47] Nierobisz LS, Felts JV, Mozdziak PE (2009). Apoptosis and macrophage infiltration occur simultaneously and present a potential sign of muscle injury in skeletal muscle of nutritionally compromised, early post-hatch turkeys. Comp Biochem Physiol B Biochem Mol Biol.

[48] Kääriäinen M, Järvinen T, Järvinen M, Rantanen J, Kalimo H (2000). Relation between myofibers and connective tissue during muscle injury repair. Scand J Med Sci Sports.

[49] Braun T, Gautel M (2011). Transcriptional mechanisms regulating skeletal muscle differentiation, growth and homeostasis. Nat Rev Mol Cell Biol.

[50] Schiaffino S, Sandri M, Murgia M (2007). Activity-dependent signaling pathways controlling muscle diversity and plasticity. Physiology (Bethesda).

[51] Qi YX, Zhang XH, Wang YQ, Pang YZ, Zhang ZB, Zhang TL, Zhang ZX (2016). Expression of MMP-1, −2, and −8 in longissimus dorsi muscle and their relationship with meat quality traits in cattle. Genet Mol Res.

[52] Weston A, Rogers R, Althen TG (2002). The role of collagen in meat tenderness. Prof Anim Sci.

[53] Schilling MW, Mink LE, Gochenour PS, Marriott NG, Alvarado CZ (2003). Utilization of pork collagen for functionality improvement of boneless cured ham manufactured from pale, soft, and exudative pork. Meat Sci.

[54] Daigle SP, Schilling MW, Marriott NG, Wang H, Barbeau WE, Williams RC (2005). PSE-like Turkey breast enhancement through adjunct incorporation in a chunked and formed deli roll. Meat Sci.

[55] Huff-Lonergan E, Lonergan SM (2005). Mechanisms of water-holding capacity of meat: the role of postmortem biochemical and structural changes. Meat Sci.

[56] Kristensen L, Purslow PP (2001). The effect of ageing on the water-holding capacity of pork: role of cytoskeletal proteins. Meat Sci.

[57] Hynes RO (1992). Integrins: versatility, modulation, and signaling in cell adhesion. Cell.

[58] van der Flier A, Sonnenberg A (2001). Function and interactions of integrins. Cell Tissue Res.

[59] Lawson MA (2004). The role of integrin degradation in post-mortem drip loss in pork. Meat Sci.

[60] Zhang WG, Lonergan SM, Gardner MA, Huff-Lonergan E (2006). Contribution of postmortem changes of integrin, desmin and μ-calpain to variation in water holding capacity of pork. Meat Sci.

[61] Oh JH, Kim YK, Jung JY, Shin JE, Kim KH, Cho KH, Eun HC, Chung JH (2011). Intrinsic aging- and photoaging-dependent level changes of glycosaminoglycans and their correlation with water content in human skin. J Dermatol Sci.

[62] Pinheiro MC, Mora OA, Caldini EG, Battlehner CN, Joazeiro PP, Toledo OM (2005). Ultrastructural, immunohistochemical and biochemical analysis of glycosaminoglycans and proteoglycans in the mouse pubic symphysis during pregnancy. Cell Biol Int.

[63] Jiang S, Jiang Z, Zhou G, Lin Y, Zheng CJJIA (2014). Effects of dietary isoflavone supplementation on meat quality and oxidative stability during storage in lingnan yellow broilers. J Integr Agr.

[64] Loeffen JL, Smeitink JA, Trijbels JM, Janssen AJ, Triepels RH, Sengers RC, van den Heuvel LP (2000). Isolated complex I deficiency in children: clinical, biochemical and genetic aspects. Hum Mutat.

[65] Smeitink J, van den Heuvel L (1999). Human mitochondrial complex I in health and disease. Am J Hum Genet.

[66] Distelmaier F, Koopman WJ, van den Heuvel LP, Rodenburg RJ, Mayatepek E, Willems PH, Smeitink JA (2009). Mitochondrial complex I deficiency: from organelle dysfunction to clinical disease. Brain.

[67] Perier C, Tieu K, Guégan C, Caspersen C, Jackson-Lewis V, Carelli V, Martinuzzi A, Hirano M, Przedborski S, Vila M (2005). Complex I deficiency primes Bax-dependent neuronal apoptosis through mitochondrial oxidative damage. Proc Natl Acad Sci U S A.

[68] Ponsuksili S, Jonas E, Murani E, Phatsara C, Srikanchai T, Walz C, Schwerin M, Schellander K, Wimmers K (2008). Trait correlated expression combined with expression QTL analysis reveals biological pathways and candidate genes affecting water holding capacity of muscle. BMC Genomics.

[69] Vargas-Galicia AJ, Sosa-Montes E, Rodríguez-Ortega LT, Pro-Martinez A, Ruiz-Feria CA, González-Cerón F, Gallegos-Sánchez J, Arreola-Enríquez J, Bautista-Ortega J (2017). Effect of litter material and stocking density on bone and tendon strength, and productive performance in broilers. Can J Anim Sci.

[70] Wu Y, Wang Y, Yin D, Wu W, Sun X, Zhang Y, Guo X, Chen J, Yuan J (2019). Effect of supplementation of nicotinamide and sodium butyrate on the growth performance, liver mitochondrial function and gut microbiota of broilers at high stocking density. Food Funct.

[71] Wu Y, Wang Y, Wu W, Yin D, Sun X, Guo X, Chen J, Mahmood T, Yan L, Yuan J (2020). Effects of nicotinamide and sodium butyrate on meat quality and muscle ubiquitination degradation genes in broilers reared at a high stocking density. Poult Sci.

[72] National Research Council (1994). Nutrient requirements of poultry.

[73] Liu Y, Yuan JM, Zhang LS, Zhang YR, Cai SM, Yu JH, Xia ZF (2015). Effects of tryptophan supplementation on growth performance, antioxidative activity, and meat quality of ducks under high stocking density. Poult Sci.

[74] Cai K, Shao W, Chen X, Campbell YL, Nair MN, Suman SP, Beach CM, Guyton MC, Schilling MW (2018). Meat quality traits and proteome profile of woody broiler breast (pectoralis major) meat. Poult Sci.

[75] Trapnell C, Pachter L, Salzberg SL (2009). TopHat: discovering splice junctions with RNA-Seq. Bioinformatics.

[76] Li B, Dewey CN (2011). RSEM: accurate transcript quantification from RNA-Seq data with or without a reference genome. BMC Bioinformatics.

[77] Robinson MD, McCarthy DJ, Smyth GK (2010). edgeR: a bioconductor package for differential expression analysis of digital gene expression data. Bioinformatics.

[78] Xie C, Mao X, Huang J, Ding Y, Wu J, Dong S, Kong L, Gao G, Li CY, Wei L (2011). KOBAS 2.0: a web server for annotation and identification of enriched pathways and diseases. Nucleic Acids Res.

