# Supplementary figures and images for: Transcriptome analysis reveals a molecular understanding of nicotinamide and butyrate sodium on meat quality of broilers under high stocking density
Source: BMC Genomics. 2020 Jun 18;21:412. doi: 10.1186/s12864-020-06827-0 (PMC7302154; doi:10.1186/s12864-020-06827-0)

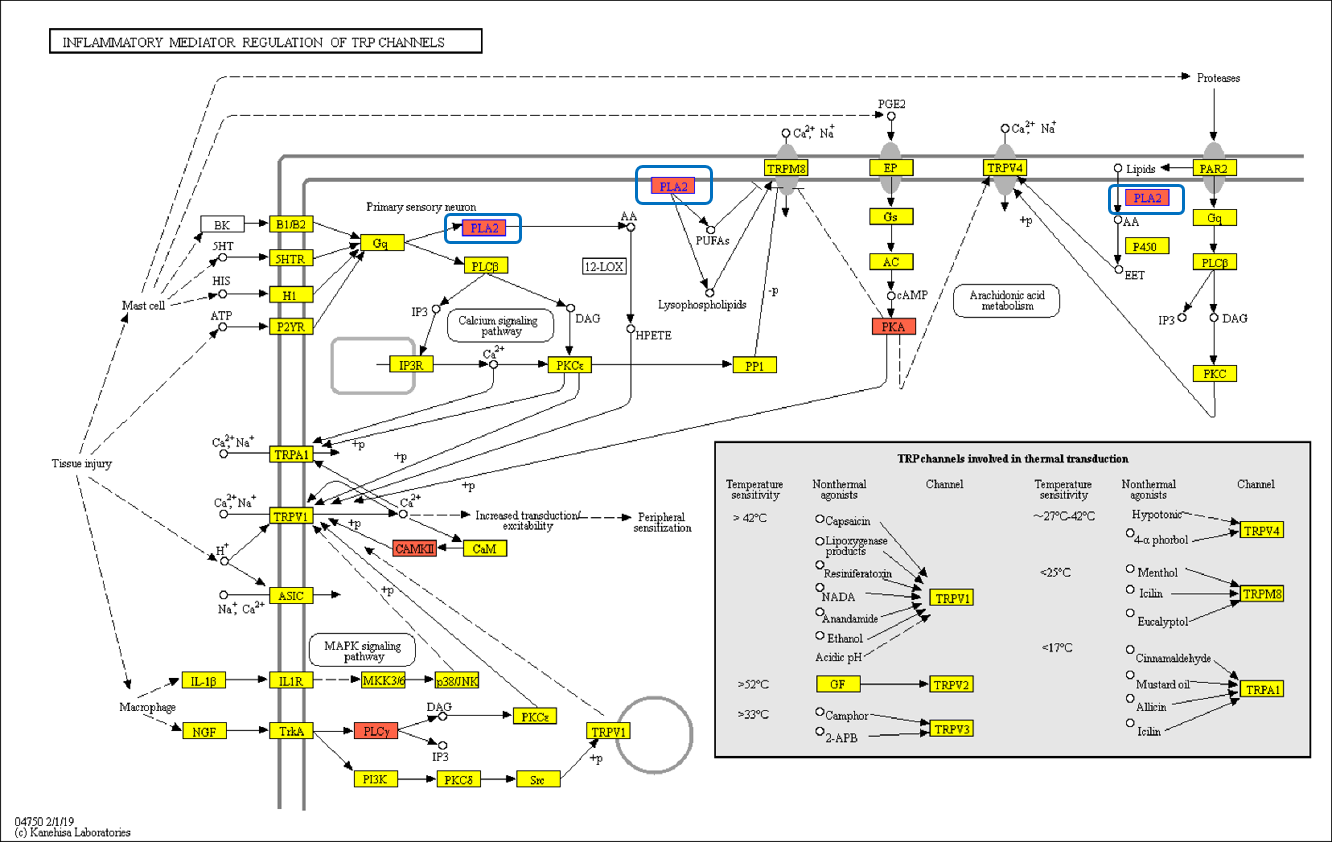

Supplement: Supplementary file 1 — Additional file 1: Figure S1. Inflammatory mediator regulation of RTP channels pathway analysis. Differential expressed genes that are involved in the inflammatory mediator regulation of RTP channels [map 04750], are highlighted. We thank Kanehisa Laboratories for providing the copyright permission of KEGG pathway maps [29]. [file 12864_2020_6827_MOESM1_ESM.tif]

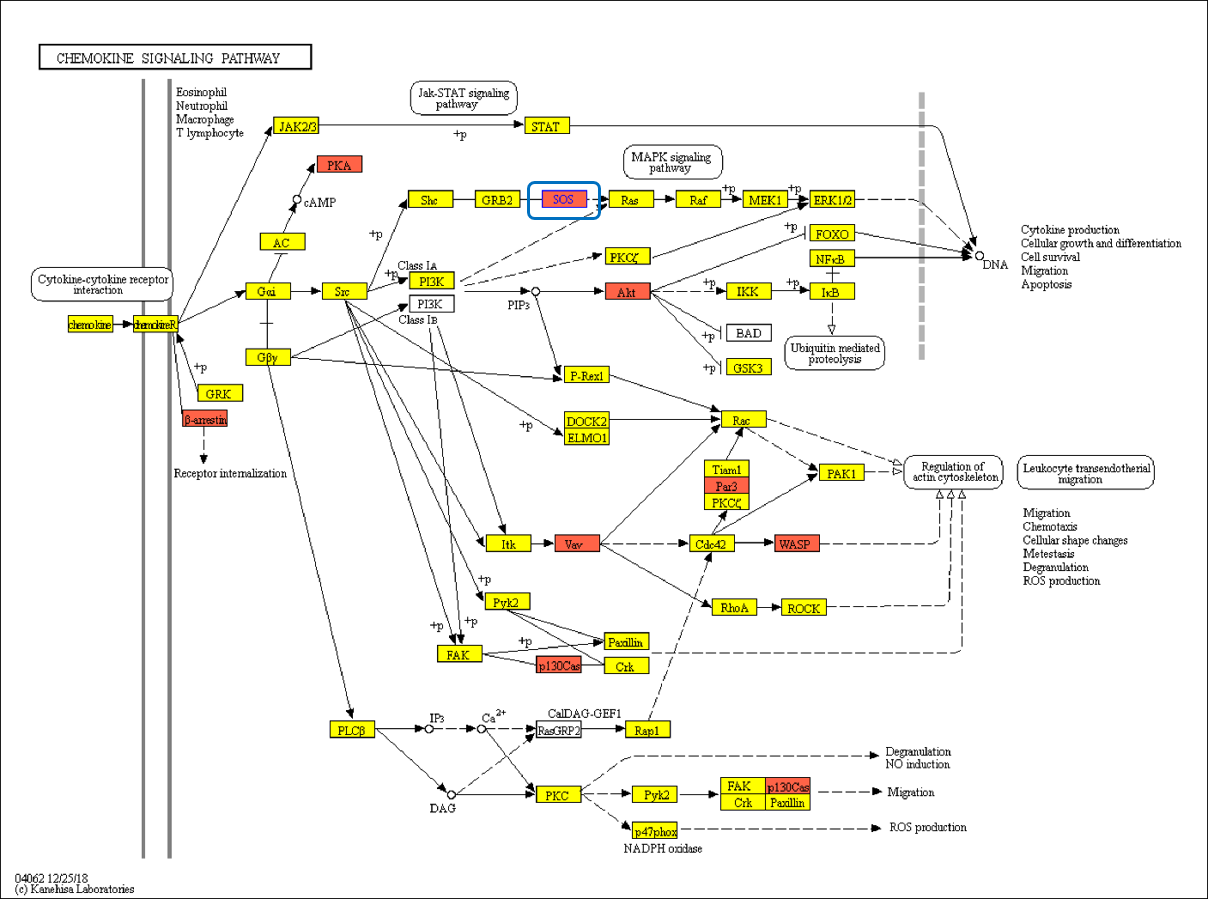

Supplement: Supplementary file 2 — Additional file 2: Figure S2. Chemokine signaling pathway analysis. Differential expressed genes that are involved in the chemokine signaling pathway [map 04062], are highlighted. We thank Kanehisa Laboratories for providing the copyright permission of KEGG pathway maps [29]. [file 12864_2020_6827_MOESM2_ESM.tif]

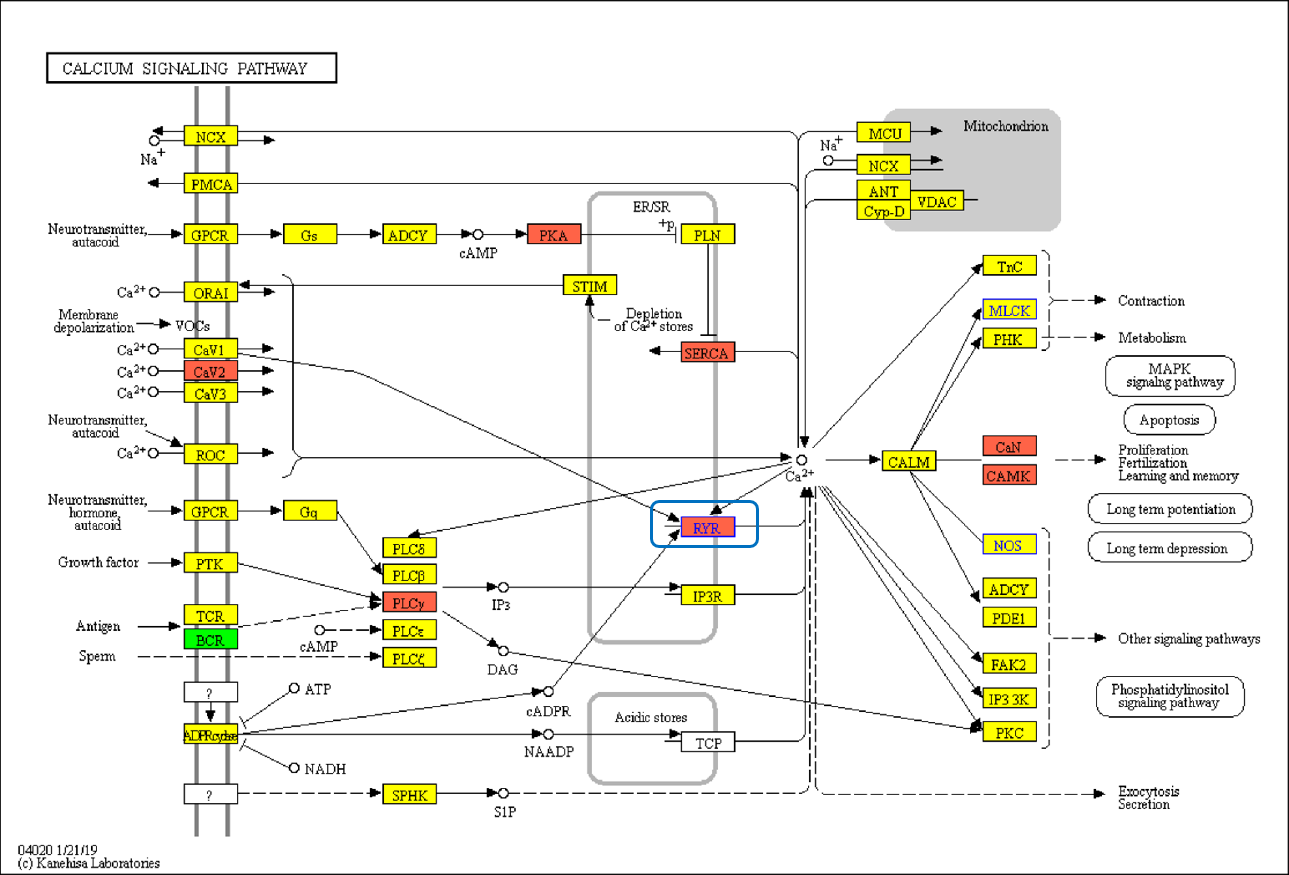

Supplement: Supplementary file 3 — Additional file 3: Figure S3. Calcium signaling pathway analysis. Differential expressed genes that are involved in the calcium signaling pathway [map 04020], are highlighted. We thank Kanehisa Laboratories for providing the copyright permission of KEGG pathway maps [29]. [file 12864_2020_6827_MOESM3_ESM.tif]

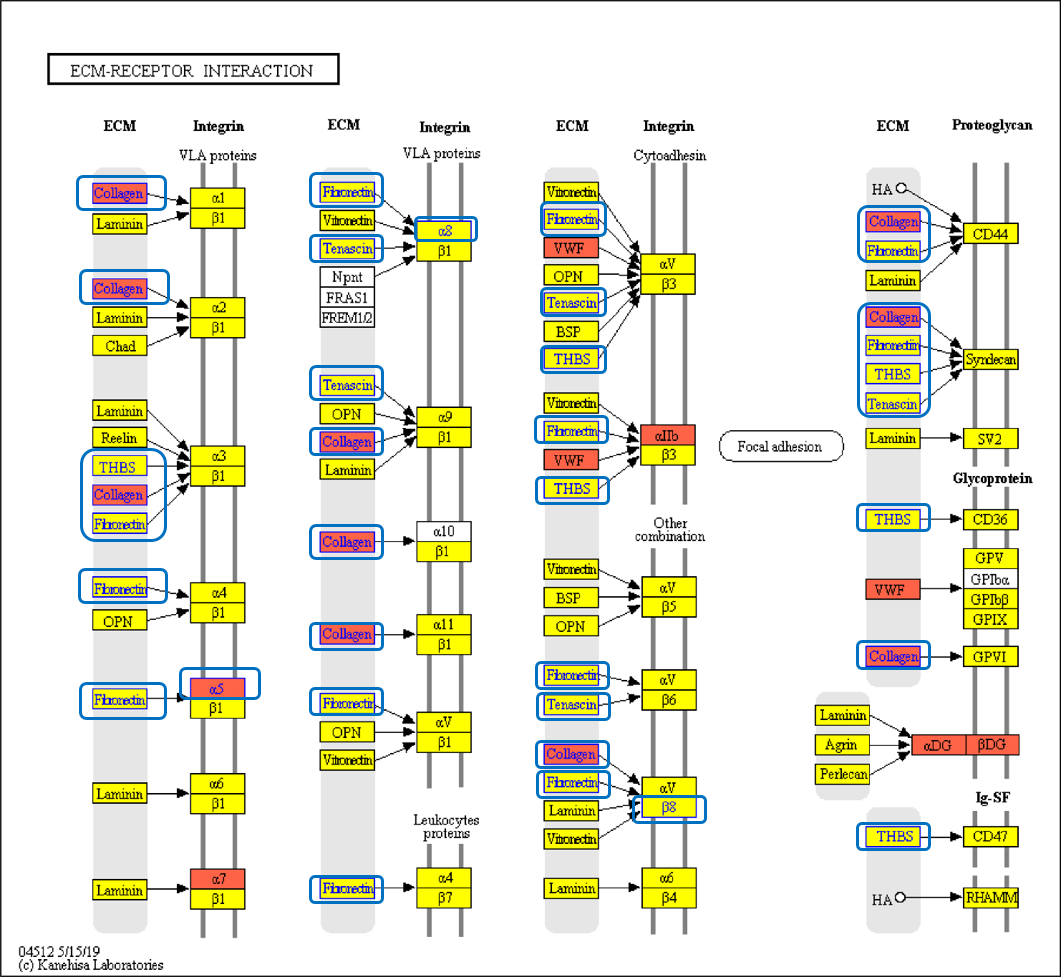

Supplement: Supplementary file 4 — Additional file 4: Figure S4. ECM-receptor interaction pathway analysis. Differential expressed genes that are involved in the inflammatory mediator regulation of RTP channels [map 04512], are highlighted. We thank Kanehisa Laboratories for providing the copyright permission of KEGG pathway maps [29]. [file 12864_2020_6827_MOESM4_ESM.tif]

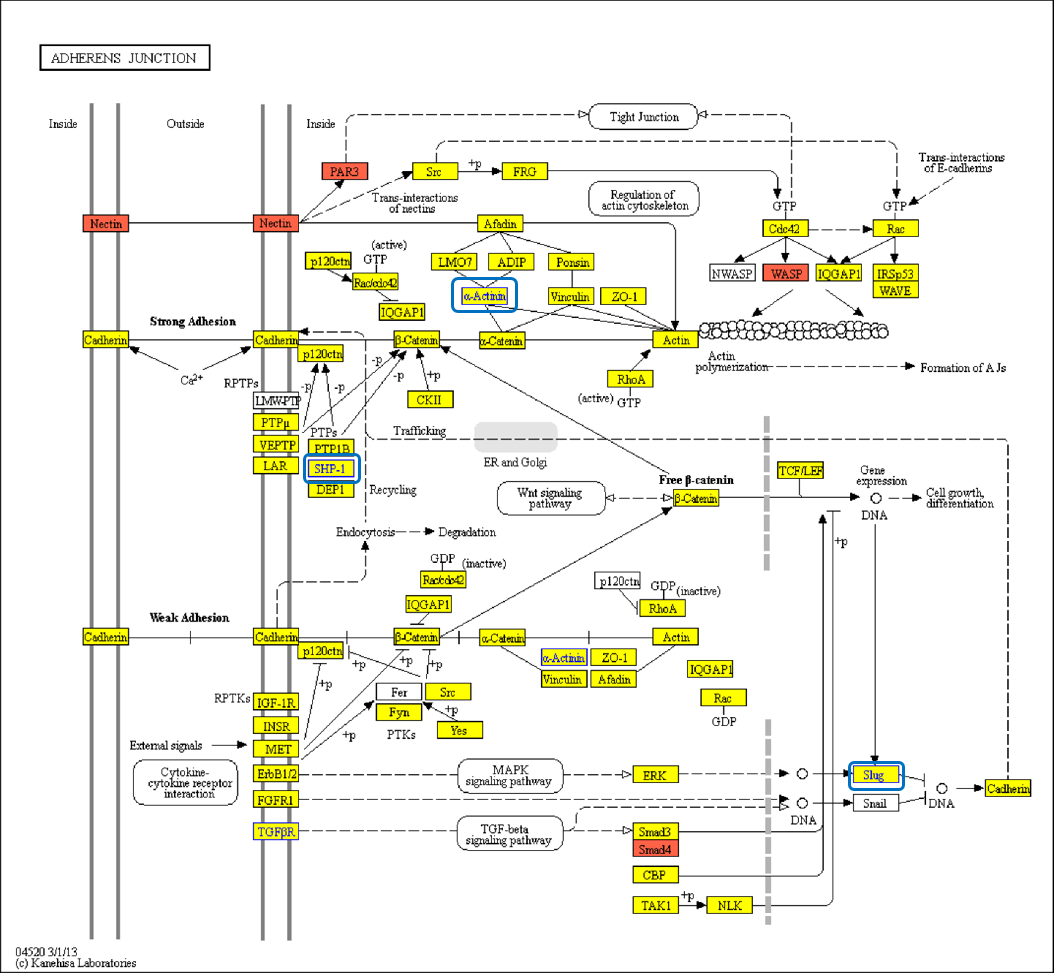

Supplement: Supplementary file 5 — Additional file 5: Figure S5. Adherens junction pathway analysis. Differential expressed genes that are involved in the adherens junction [map 04520], are highlighted. We thank Kanehisa Laboratories for providing the copyright permission of KEGG pathway maps [29]. [file 12864_2020_6827_MOESM5_ESM.tif]

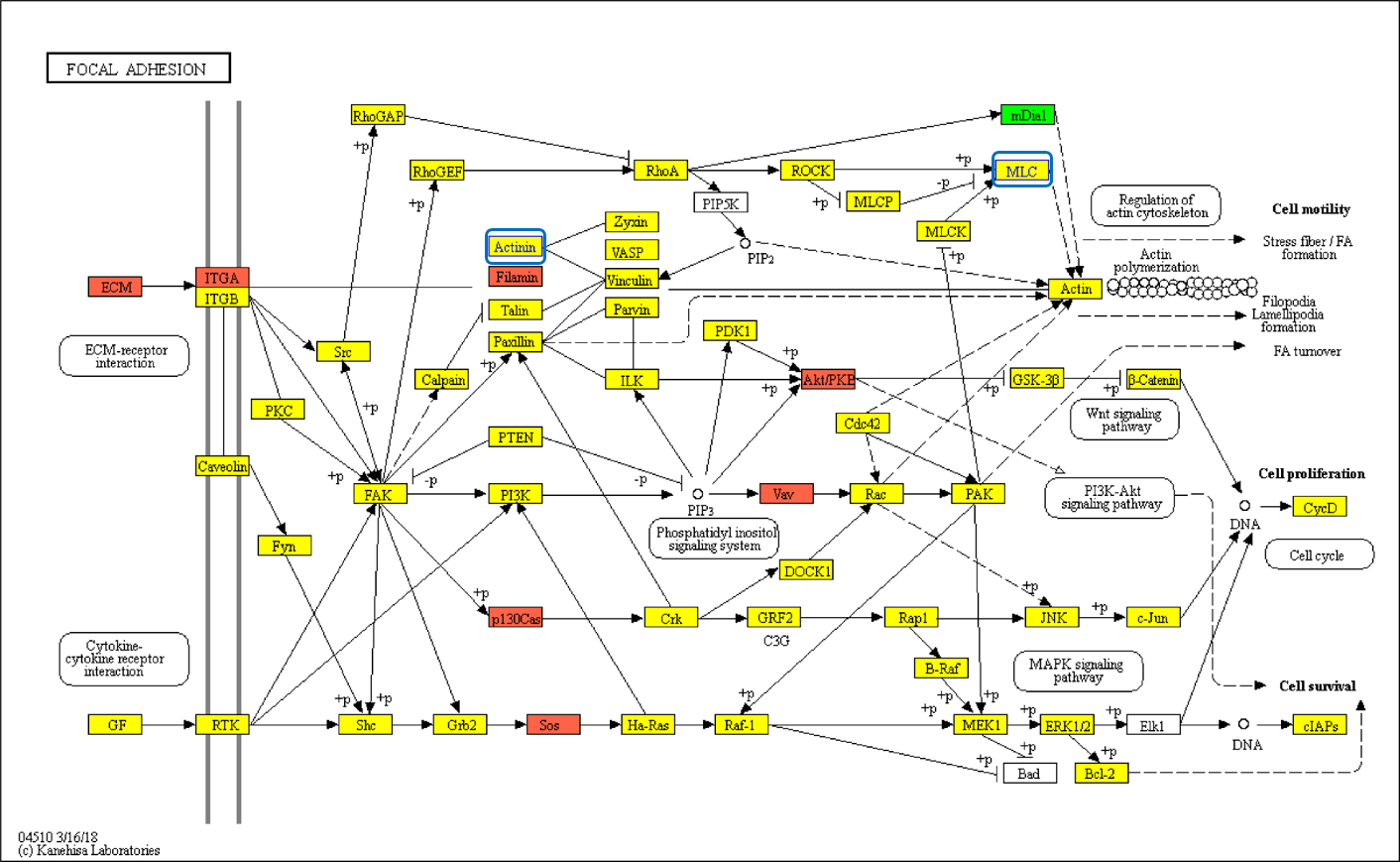

Supplement: Supplementary file 6 — Additional file 6: Figure S6. Focal adhesion pathway analysis. Differential expressed genes that are involved in the focal adhesion [map 04510], are highlighted. We thank Kanehisa Laboratories for providing the copyright permission of KEGG pathway maps [29]. [file 12864_2020_6827_MOESM6_ESM.tif]

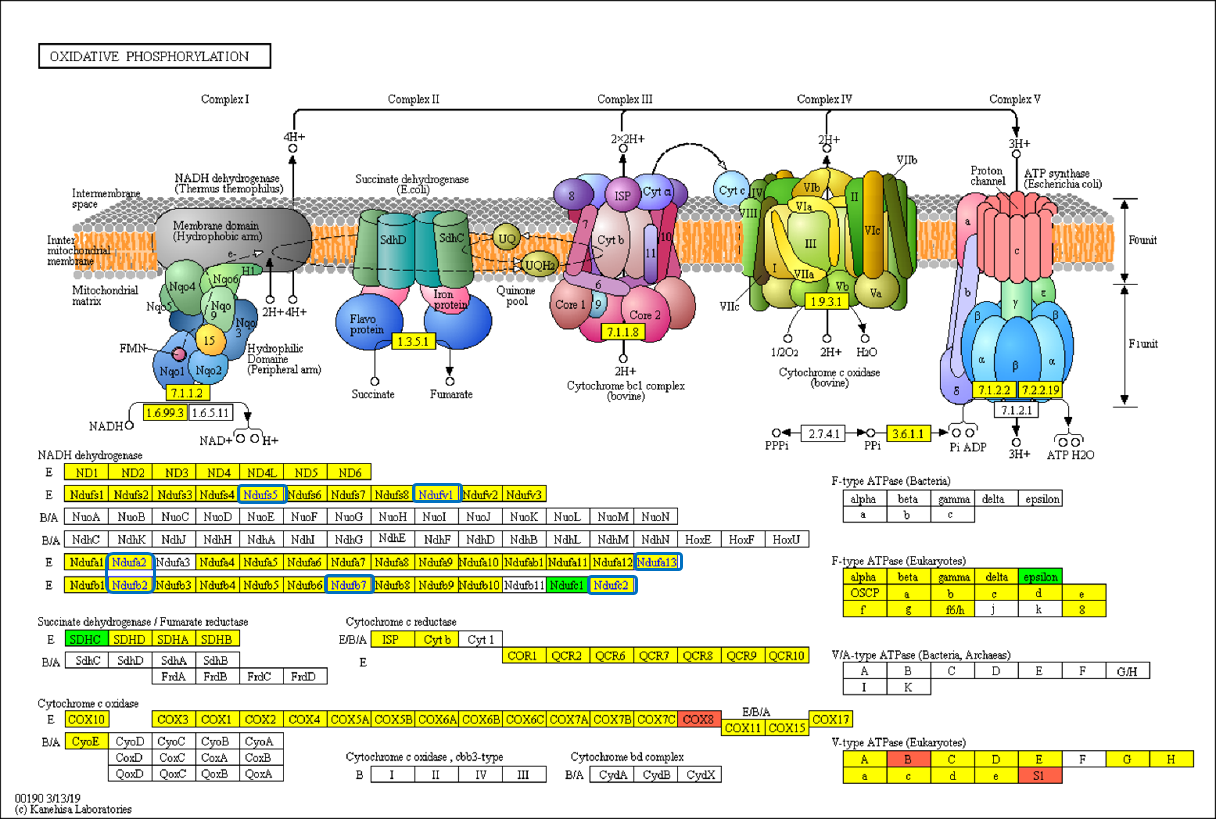

Supplement: Supplementary file 7 — Additional file 7: Fig. S7. Oxidative phosphorylation pathway analysis. Differential expressed genes that are involved in the oxidative phosphorylation [map 00190], are highlighted. We thank Kanehisa Laboratories for providing the copyright permission of KEGG pathway maps [29]. [file 12864_2020_6827_MOESM7_ESM.tif]

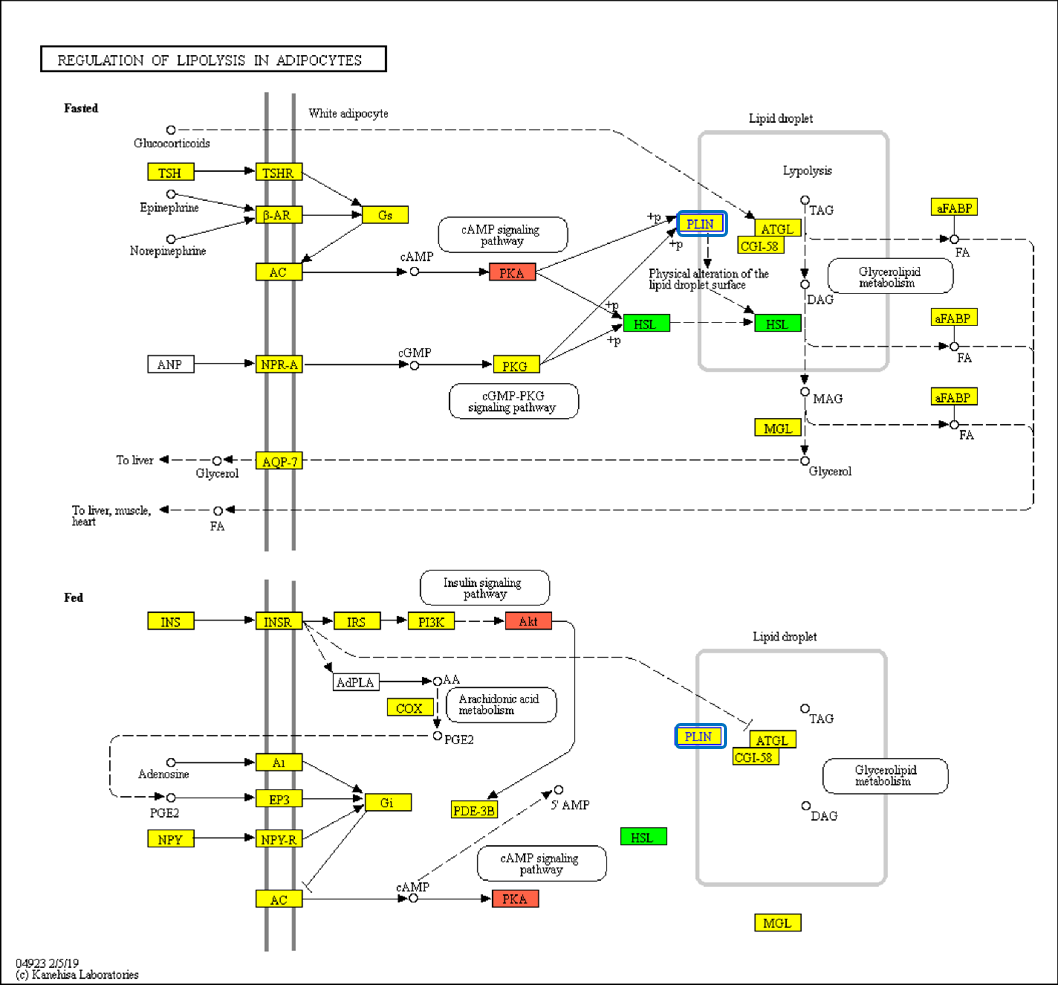

Supplement: Supplementary file 8 — Additional file 8: Figure S8. Regulation of lipolysis in adipocytes pathway analysis. Differential expressed genes that are involved in the Regulation of lipolysis in adipocytes [map 04923], are highlighted. We thank Kanehisa Laboratories for providing the copyright permission of KEGG pathway maps [29]. [file 12864_2020_6827_MOESM8_ESM.tif]

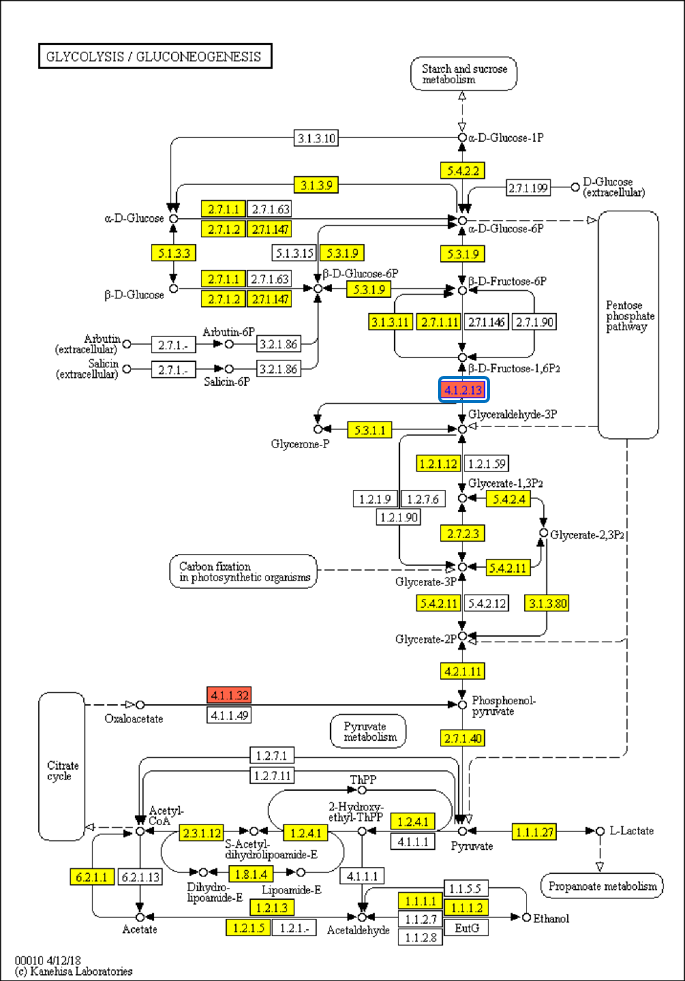

Supplement: Supplementary file 9 — Additional file 9: Figure S9. Glycolysis/Gluconeogenesis pathway analysis. Differential expressed genes that are involved in the Glycolysis/Gluconeogenesis [map 00010], are highlighted. We thank Kanehisa Laboratories for providing the copyright permission of KEGG pathway maps [29]. [file 12864_2020_6827_MOESM9_ESM.tif]

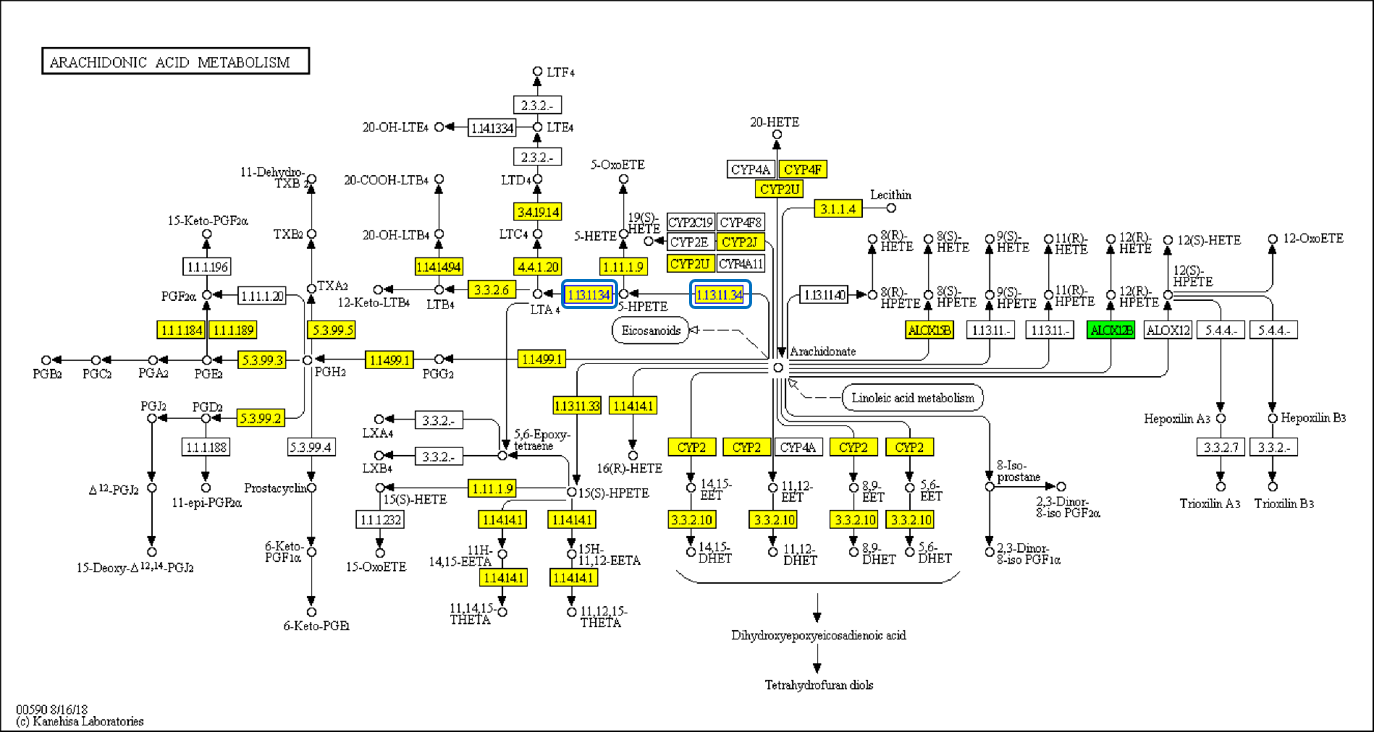

Supplement: Supplementary file 10 — Additional file 10: Figure S10. Arachidonic acid metabolism pathway analysis. Differential expressed genes that are involved in the arachidonic acid metabolism [map 00590], are highlighted. We thank Kanehisa Laboratories for providing the copyright permission of KEGG pathway maps [29]. [file 12864_2020_6827_MOESM10_ESM.tif]
